# Antimicrobially Active Zn(II) Complexes of Reduced Schiff Bases Derived from Cyclohexane-1,2-diamine and Fluorinated Benzaldehydes—Synthesis, Crystal Structure and Bioactivity

**DOI:** 10.3390/life13071516

**Published:** 2023-07-06

**Authors:** Bianka Oboňová, Ladislav Habala, Miroslava Litecká, Peter Herich, Andrea Bilková, František Bilka, Branislav Horváth

**Affiliations:** 1 Department of Chemical Theory of Drugs, Faculty of Pharmacy, Comenius University in Bratislava, Odbojárov 10, 832 32 Bratislava, Slovakia; obonova@fpharm.uniba.sk (B.O.); herich@fpharm.uniba.sk (P.H.); 2 Department of Materials Chemistry, Institute of Inorganic Chemistry of the CAS, Husinec-Řež č.p. 1001, 250 68 Řež, Czech Republic; litecka@iic.cas.cz; 3 Department of Physical Chemistry, Faculty of Chemical and Food Technology, Slovak University of Technology, Radlinského 9, 812 37 Bratislava, Slovakia; 4 Department of Cellular and Molecular Biology of Drugs, Faculty of Pharmacy, Comenius University in Bratislava, Odbojárov 10, 832 32 Bratislava, Slovakia; bilkova@fpharm.uniba.sk (A.B.); bilka@fpharm.uniba.sk (F.B.); 5 NMR Laboratory, Faculty of Pharmacy, Comenius University in Bratislava, Odbojárov 10, 832 32 Bratislava, Slovakia; horvath@fpharm.uniba.sk

**Keywords:** antimicrobial activity, metal complexes, fluorinated compounds, medicinal chemistry, Schiff bases, zinc

## Abstract

A series of Schiff base ligands obtained by the condensation of *trans*-cyclohexane-1,2-diamine and fluorinated benzaldehydes were prepared, followed by their reduction with NaBH_4_. The reduced ligands were employed in the synthesis of zinc complexes of the general formula [ZnCl_2_(L)]. The structures of both the original and the reduced Schiff bases, as well as of the zinc complexes, were characterized by single-crystal X-ray analysis, along with NMR and IR spectroscopy. The antimicrobial activities of the reduced Schiff bases and their zinc complexes were evaluated in vitro against *E. coli*, *S. aureus*, and *C. albicans*. The compounds containing the 4-(trifluoromethylphenyl) moiety showed marked antibacterial activity. Interestingly, the antimicrobial effect of the zinc complex with this moiety was significantly higher than that of the corresponding free reduced ligand, comparable with ciprofloxacin used as standard. Thus, a synergic effect upon the complexation with zinc can be inferred.

## 1. Introduction

The advent of modern antimicrobial chemotherapy at the beginning of the 20th century meant a breakthrough in medicine and led to a radical decrease in mortality due to communicable diseases. Interestingly, inorganic (or organometallic) compounds were among the first antimicrobial drugs in clinical use (e.g., arsphenamine, antimonials). Later, though, they were largely superseded by efficient organic antibiotics. However, in recent years, there has been a renaissance of interest in metal-based antimicrobials as the established antibiotics are increasingly susceptible to antimicrobial resistance, posing a major threat to public health [1]. Metal complexes offer distinct mechanisms of biological action, potentially leading to more potent antimicrobial drugs [2,3]. Nevertheless, the biological activity of metal-containing compounds is still insufficiently understood, necessitating further research into this area.

Schiff bases and their metal complexes represent a unique category of compounds with numerous interesting applications, e.g., in medicinal chemistry, catalysis, environmental technology or industrial chemistry [4,5,6,7]. Schiff bases are a type of ligands widely used in coordination chemistry because of their simple preparation, remarkable variety of structural arrangements, coordination abilities, and possible biological effects. There are numerous studies describing the biological activities of Schiff bases and their metal complexes, many of them exhibiting antimicrobial, anticancer, antiviral, anti-inflammatory, or enzyme-inhibitory properties (e.g., [8,9,10,11]). Distinctive biological activities are exerted by Schiff bases containing heterocyclic moieties (e.g., [12,13]).

There are several types of Schiff bases, differing in structural characteristics and biological activities. An important category are the hydrazones, which are Schiff bases with hydrazine or its organic derivatives [14,15]. Another prominent member of the Schiff base class are the thiosemicarbazones, derivatives of thiosemicarbazide with marked anticancer activities [16,17,18]. A successful example is triapine, 3-aminopyridine-2-carbaldehyde thiosemicarbazone, a potent ribonucleotide reductase inhibitor and a very strong iron chelator, currently undergoing clinical trials [19,20]. Interestingly, coordination of triapine and related compounds with copper ions modulates their physicochemical properties and anticancer activity [21].

Coordination of Schiff bases with metal ions often enhances their biological activity [22]. However, one drawback of these compounds is the hydrolysis of the azomethine group in the presence of water molecules. This can be overcome by reducing the imine to an amine group, making the structure more stable and less rigid upon coordination with a metal centre. Using diamines (or, alternatively, dicarbonyl compounds) as starting materials leads to polydentate ligands (bis-Schiff bases) with excellent chelating properties. Typical representatives of such compounds are SALEN-type ligands, a versatile category of ligands with manifold applications [23]. An important starting compound for their synthesis is *trans*-1,2-cyclohexanediamine, used as a ligand in the anticancer drug oxaliplatin.

In continuation of our previous research on bioactive Schiff base metal complexes [24,25], we present here the preparation, structural elucidation, and preliminary evaluation of the antimicrobial potency in vitro of zinc complexes with reduced Schiff base ligands. Zinc is a biogenic element, possessing good coordinating ability and relatively low toxicity. Its complexes also exhibit marked antimicrobial effects [26,27,28]. We have chosen fluorine-containing moieties for the synthesis of Schiff base ligands since fluorinated compounds represent a category of molecules with specific pharmacological properties [29]. The ligands were prepared according to established synthetic procedures. The products (zinc complexes and selected ligands) were screened for their antimicrobial properties, with the aim of evaluating the impact of the type and location of the substituents on the bioactivity of the synthesized compounds. Besides the structures of the zinc complexes, we also report the X-ray single-crystal data of the Schiff bases, being of considerable importance in evaluating diverse pharmacological parameters. The results form the basis for further investigation of this class of compounds.

Several complexes of reduced Schiff bases from substituted benzaldehydes and 1,2-diaminocyclohexane have been synthesized and their X-ray crystal structures described in the literature. They have the general structure [Zn(L)Cl_2_]. The structure of an analogous complex with chiral *N*,*N′*-dibenzyl-(*R*,*R*)-1,2-diaminocyclohexane has been reported [30]. The complex is chiral due to the presence of an enantiomerically pure ligand. Similar to the complexes prepared by us, the coordination in the zinc complex is distorted tetrahedral. A related structure shows the Zn(II) complex with the reduced Schiff base *N*,*N′*-bis(4-methoxy-benzyl)-cyclohexane-1,2-diamine [31]. Dichloro zinc complexes with *N*,*N′*-bis-(2,6-dichloro-benzyl)-(*R*,*R*)-1,2-diaminocyclohexane and with *N*,*N′*-bis(2-fluorobenzyl)-(*R*,*R*)-1,2-diaminocyclohexane have also been described, both exhibiting nearly tetrahedral geometry [32,33]. Reduced Schiff base Zn(II) complexes of cyclohexane-1,2-diamine containing naphthalene moieties were synthesized [34]. Along with the normal bis-Schiff base complexes, a zinc complex with an unsymmetrical Schiff base (only one amino group substituted) was also prepared.

## 2. Materials and Methods

### 2.1. General

All chemicals used in the syntheses were purchased from Sigma-Aldrich (St. Louis, MO, USA) and used as received, without further purification. Nicolet 6700 FT-IR spectrometer (manufacturer Thermo Scientific, Waltham, MA, USA) was employed for the recording of infrared spectra. The instrument uses the Attenuated Total Reflectance method (ATR) for the measurement. The spectrometer operated within the wavenumber range of 600–4000 cm^−1^. Flash2000 elemental analyser (manufacturer Thermo Scientific) was utilized to establish the elemental composition (C, H, N) of the synthetic products. ^1^H- and ^13^C-NMR spectra were registered on a Varian MR400 NMR spectrometer using DMSO-*d*_6_, CD_3_OD, and CDCl_3_ as solvents and TMS (tetramethylsilane) as the internal standard. Chemical shifts were expressed as δ values, and the coupling constants (*J*) were given in Hz. The measurement of melting points was carried out using a Büchi B-540 Melting Point Determinator (manufacturer Büchi Labortechnik AG, Flawil, Switzerland).

### 2.2. Synthesis of Schiff Bases and Reduced Schiff Bases

#### 2.2.1. General Synthetic Procedure for the Preparation of Schiff Bases

To a solution of *trans*-1,2-cyclohexanediamine in methanol, 4-fluorobenzaldehyde was added dropwise. The mixture was stirred at room temperature for 2 h and left to crystallize freely at room temperature. After several days, colorless crystals were filtered and washed with cold ethanol. Crystals suitable for X-ray structure analysis were obtained by recrystallisation from ethanol.

#### 2.2.2. General Synthetic Procedure for the Preparation of Reduced Schiff Bases

To a solution of a Schiff base in methanol, NaBH_4_ was added and the mixture was stirred for several hours at room temperature until the yellow color disappeared. The solvent was evaporated to dryness and water was added to the residue. The product was extracted with chloroform (3 × 15 mL) and dried over anhydrous sodium sulphate. The pH of the solution was set to 4–5 by adding 3M HCl. The white solid precipitate was filtered and washed with cold water, ethanol, and diethyl ether. The product was recrystallized from ethanol and characterized by X-ray structure analysis.

Yields and characterisation data for the ligands are summarized in Table 1. More details about the syntheses of the Schiff bases and their reduced forms, along with their analytical characterization, can be found in Supplementary Materials.

### 2.3. Synthesis of the Zinc Complexes

The reduced Schiff base ligand was dissolved in ethanol and then a solution of the corresponding metal salt (ZnCl_2_) in ethanol was added under constant stirring. The mixture was heated to 50 °C and stirred for 3 h, after which it was left to cool off to room temperature. After several hours, the precipitate was collected and washed with distilled water and ethanol and dried in vacuum. The product was recrystallized from ethanol/acetonitrile to obtain crystals suitable for X-ray structure analysis. Yields and characterisation data are summarized in Table 1. More details about the syntheses of the zinc complexes and their analytical characterization can be found in Supplementary Materials.

### 2.4. X-ray Structure Determination

Diffraction measurements for the compounds **L1**, **L2**, and **L4** were carried out with the help of a Stoe STADIVARI diffractometer. Dectris Pilatus 300 K detector together with a Genix3D Cu HF source were used (λ = 1.54186 Å, Cu-Kα). The temperature was kept at 100 K during the experiment employing a Cobra Oxford Cryosystems cooler with a nitrogen gas open flow. Stoe X-Area detector software was applied for data reduction [35]. The refinement for all non-hydrogen atoms was done with anisotropic thermal parameters. Optimization of positions of all H atoms was carried out under the constrain of riding on their parent atoms, with C–H (aromatic and imino group) bond length of 0.95 Å (for all compounds **L1**, **L2**, and **L4**), C–H(aliphatic) bond length of 0.99 Å, and C–H(aliphatic asymetric) bond length of 1.00 Å. Hydrogen’s temperature factors Uiso(H) = 1.2 Ueq(C).

Data for the remaining monocrystals (except **L1**, **L2**, and **L4**) was collected on the Rigaku XtaLAB Synergy S diffractometer equipped with micro-focus CuKα radiation and a Hybrid Pixel Array Detector (HyPix-6000HE). An Oxford Cryosystems (Cryostream 800) cooling device was used for data collection and the crystals were kept at 100 K during data collection. The collection and reduction of data as well as cell refinement were accomplished using CrysAlisPro software [36]. The correction of data for absorption effects was done with the help of the empirical absorption correction method (i.e., spherical harmonics) as a part of the scaling algorithm SCALE3 ABSPACK. Numerical absorption correction was employed, relying on gaussian integration over the multifaceted crystal model. Hydrogen atoms of all molecules were placed in calculated positions.

The crystal structures of all compounds were solved in the OLEX2 software [37] using the SHELXT-2015 program via Intrinsic Phasing [38] and refined with SHELXL-2015 by the least-squares procedure on F^2^ [39]. The DIAMOND program (version 2.1e) [40] was used for the molecular graphics.

### 2.5. Antimicrobial Activity

Antimicrobial activities of the synthesized Schiff bases and their zinc(II) complexes were evaluated against Gram-positive and Gram-negative bacteria and against yeast, namely the strains *Staphylococcus aureus* CNCTC Mau 29/58 and *Escherichia coli* CNCTC 377/79 (both purchased from the Czech National Collection of Type Cultures, Czech Republic), and *Candida albicans* CCM 8186 (from the Czech Collection of Microorganisms, Czech Republic).

The determination of minimum inhibitory concentration (MIC) was done via the broth dilution method as described in [41]. Stock solutions with initial concentrations 45 mM for ligands and 27 mM for zinc complexes were prepared in DMSO immediately before use. For comparison, ciprofloxacine was used as a standard compound.

## 3. Results and Discussion

### 3.1. Synthesis

**Scheme 1:**
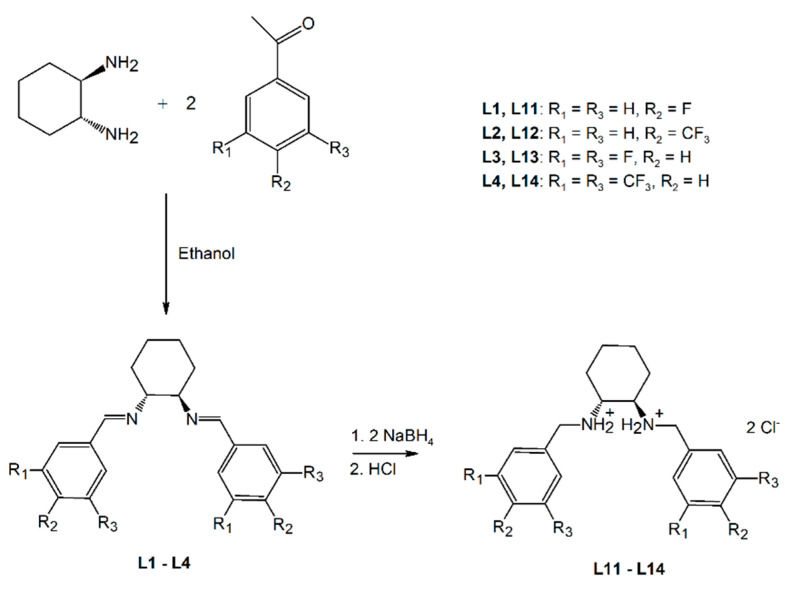
Synthesis of Schiff bases **L1**–**L4** and their reduced forms **L11**–**L14**.

The Schiff bases **L1**–**L4** were prepared by condensation reactions of 1 equiv. of 1,2-cyclohexanediamine and 2 equiv. of fluorinated benzaldehydes in high yields (>70%). This direct method (carried out in methanol) provided X-ray quality transparent crystals. If further purification is needed, products may be recrystallized from an ethanolic solution. Reduction of imine bonds in the prepared Schiff bases with sodium borohydride gave substituted amines **L11**–**L14** obtained in moderate yields (Scheme 1). All products are stable in the air and soluble in most organic solvents, such as EtOH, MeOH, CHCl_3_, etc.

**Figure 1:**
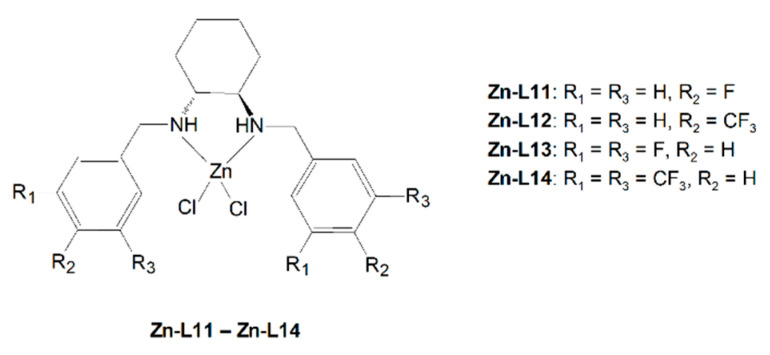
Chemical structure of **Zn-L11**–**L14** complexes.

Treatment of the reduced Schiff base ligands with ZnCl_2_∙4H_2_O in an 1:1 ratio led to the respective zinc(II) complexes (Figure 1). Products were recrystallized from an ethanol/acetonitrile medium and the collected white monocrystals analyzed. The complexes exhibit limited solubility in most organic solvents. Moderate solubility can be achieved with dimethylformamide (DMF) and dimethyl sulfoxide (DMSO). Structures of ligands and complexes were characterized with appropriate analytical methods. The complexes are stable in DMSO solution, as proven by measuring their NMR spectra within three days.

### 3.2. Analytical Characterization

Elemental analysis of all products corresponds to their assumed structure, as can be seen in the Materials and Methods section. For the measurement of ^1^H and ^13^C-NMR spectra of Schiff bases, deuterated solvents were used—CD_3_Cl for **L1**–**L4** and CD_3_OD for **L11**–**L14**. The imine groups in Schiff bases **L1**–**L4** appear as singlets at δ 8.08–8.27 ppm. These singlets completely disappear in the spectra of their reduced forms and new doublets in the range 4.29–4.64 ppm appear, which refer to protons in the Ar-CH_2_-N^+^H_2_- group. The peaks of the Ar-CH_2_-NH_2_- group are visible around 3.96–3.75 ppm but the protonation of the neighbouring amino group shifted these peaks to higher ppm [42]. Aromatic ring protons are observed in the range of 7–8 ppm and the cyclohexane CH/CH_2_ groups appear approximately at 1–3 ppm.

In infrared spectra of the ligands, vibration signals of the imine group ν(CH=NH) are visible as sharp peaks at 1643–1652 cm^−1^. The absence of these peaks and new broad vibration signals at 2400–2700 cm^−1^ indicate the disappearance of these imine groups in the course of reduction and the creation of new protonated ammonium groups. The strong sharp peak near 1100–1300 cm^−1^ refers to C-F bond vibration.

Coordination to a metal ion shifts the stretching frequency of donor groups to lower cm^−1^. Absorption bands of the -NH- group after coordination appear at 3206–3189 cm^−1^, which is lower than the vibration bands of free -NH- at 3320–3280 cm^−1^ [43].

### 3.3. X-ray Crystallography

**Figure 2:**
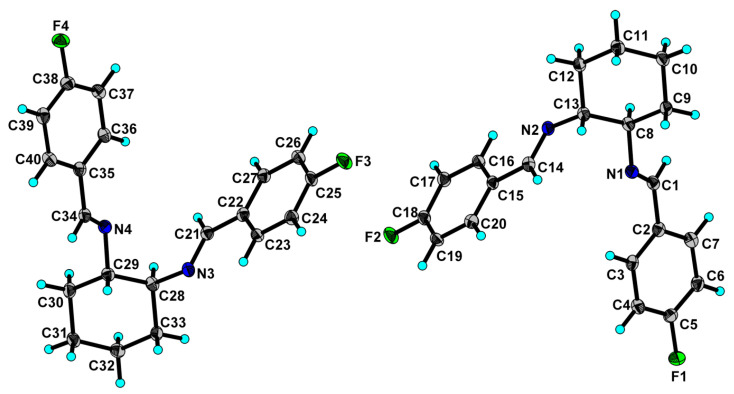
The X-ray crystal structure of **L1** representing the atom-labelling scheme. Displacement ellipsoids are depicted at 50% probability level.

**Figure 3:**
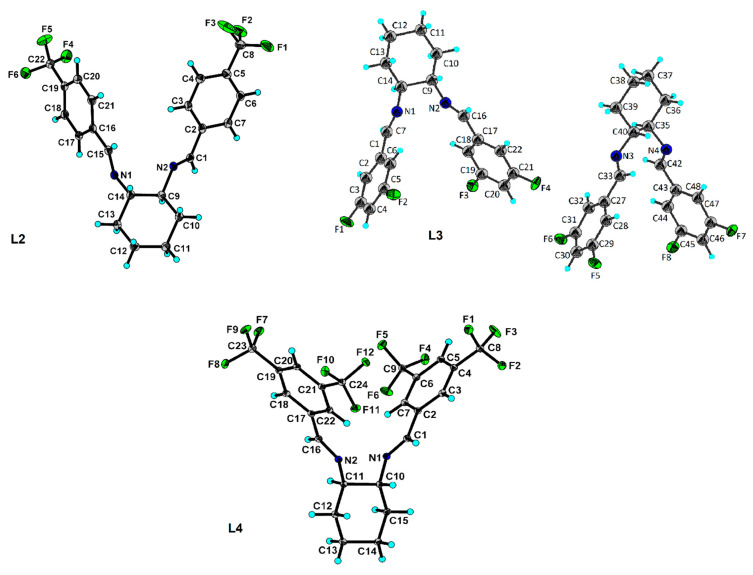
The X-ray crystal structure of **L2**–**L4** displaying the atom-labelling scheme. Displacement ellipsoids are depicted at 50% probability level.

The compounds **L1**, **L2** crystallized in the orthorhombic space groups–Pna2_1_ (No. 33) for **L1**, and Pca2_1_ (No. 29) for **L2**. The compound **L3** crystallized in the monoclinic P2_1_/c (No. 14) and the compound **L4** in the triclinic P-1 space group (No. 2). The compound **L1** crystallized as two independent molecules in an asymmetric unit, with eight molecules in the unit cell. The other compounds (**L2**–**L4**) crystallized as one independent molecule in an asymmetric unit with four molecules (for **L2**) and two molecules (for **L4**) in the unit cell. The molecular structures for compounds (**L1**–**L4**) are shown in Figure 2 and Figure 3 and their crystallographic data are summarized in Table S4. The crystal structures of compounds **L1**–**L4** are stabilized by a network of intermolecular hydrogen bonds and interactions (Table S1, Figures S1–S4 in Supplementary Materials).

**Figure 4:**
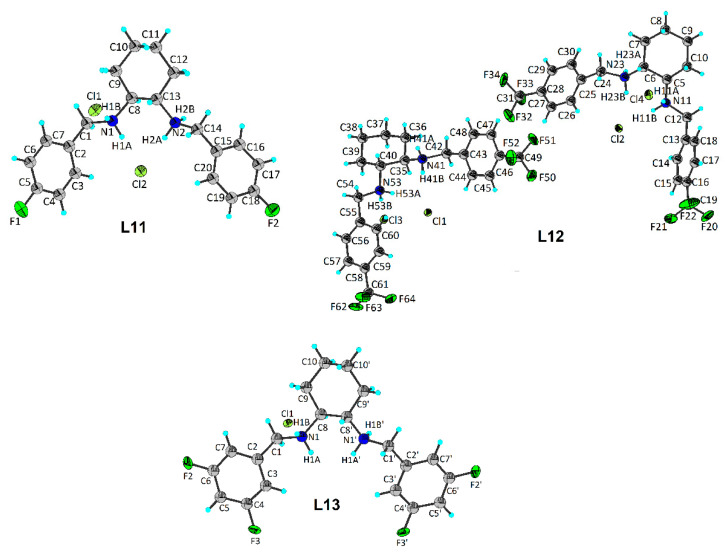
The X-ray crystal structures of **L11**–**L13** displaying the atom-labelling scheme. Displacement ellipsoids are depicted at 50% probability level.

The Schiff bases **L1**–**L4** were reacted with NaBH_4_ to yield reduced Schiff bases **L11**–**L14**. The compounds were isolated in the form of their salts with HCl and, for the compounds **L11**–**L13**, white or colorless crystals suitable for single-crystal X-ray analysis could be prepared by slow evaporation from the ethanolic solution. The compound **L11** crystallized in the tetragonal I4_1_/a space group (No. 88), the compounds **L12** and **L13** in the orthorhombic space groups–P2_1_2_1_2_1_ (No. 19) for **L12,** and P*bcn* (No. 60) for **L13**. All compounds are hydrochlorides, i.e., the crystal structures contain chloride counterions compensating the positive charge on the amino groups. There are two chloride ions for each molecule, indicating that both amino groups are protonated. **L12** crystallized as two independent molecules in an asymmetric unit, with eight molecules in the unit cell. Compounds **L11** and **L13** crystallized each as one independent molecule in an asymmetric unit, with 16 (**L11**) and 4 (**L13**) molecules in the unit cell. The molecular structures for compounds (**L11**–**L13**) are shown in Figure 4 and their crystallographic data are summarized in Table S5. The crystal structures of compounds **L11**–**L13** are stabilized by a system of intermolecular hydrogen bonds and interactions (Table S2, Figures S5–S7 in Supplementary Materials).

**Figure 5:**
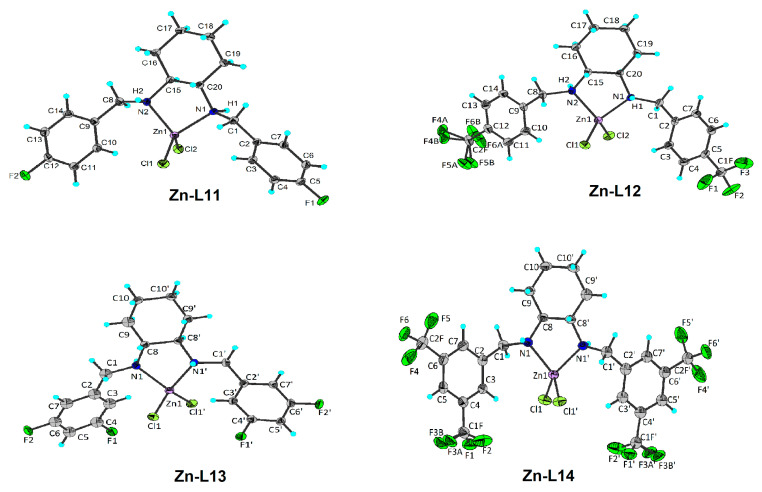
The X-ray crystal structures of **Zn-L11**–**Zn-L14** displaying the atom-labelling scheme. Displacement ellipsoids are depicted at 50% probability level.

The reduced Schiff bases were employed in the synthesis of zinc complexes (**Zn-L11**–**Zn-L14**). White monocrystals were prepared by the recrystallization of the complexes from ethanol/acetonitrile. Compound **Zn-L11** crystallized in the orthorhombic P*bca* space group (No. 61), and compound **Zn-L12** in the triclinic *P*-1 (No. 1) group. Both **Zn-L13** and **Zn-L14** complexes crystallized in the monoclinic space group C2/c (No. 15). The molecular structures for compounds **Zn-L11**–**Zn-L14** are shown in Figure 5 and their crystallographic data are summarized in Table S6. The crystal structures of the complexes **Zn-L11**–**Zn-L14** are stabilized by a system of intermolecular hydrogen bonds and interactions (Table S3, Figures S8–S11 in Supplementary Materials).

Selected bond lengths (Å) and angles (°) for the zinc complexes are given in Table 2. The complexes are mononuclear with distorted tetrahedral geometry and tetra-coordinate central atoms. In all complexes, the coordination of the chloride ligands is retained; thus, the overall charge of the complexes is zero and the complexes are non-ionic. The reduced Schiff base ligands coordinate with the central zinc atom through both nitrogen atoms. The bond lengths between the zinc atoms and nitrogen atoms are almost identical (about 2.07 Å), except for the complex **Zn**–**L11**, where one Zn-N bond is significantly shorter. The same pattern applies to bond lengths between the zinc atoms and chloride ligands, which amount in most cases to 2.22–2.23 Å. The length of the Zn–Cl contact is within a typical range for this type of complexes. The bond angles exhibit strong distortion in comparison to the ideal tetrahedral bond angle, with the minimum being reached in N1–Zn–N2 (86.7–88.0 Å) and the maximum in Cl1–Zn–Cl2 (117.3–120.1 Å; except in **Zn**–**L14** where the highest bond angle was encountered in N–Zn–Cl).

### 3.4. Biological Activity

Antimicrobial activities of zinc complexes (**Zn-L11**–**Zn-L14**) were evaluated in vitro against Gram-negative (*E. coli*) and Gram-positive (*S. aureus*) bacterial strains and against the yeast *C. albicans.* The activities of representative starting compounds (**L1**, **L11**, **L12**) were assessed for comparison. Minimal inhibition concentrations (MIC) of the compounds are shown in Table 3 along with ciprofloxacine used as a standard. The inhibition activities of the compounds against Gram-negative strains are either comparable to the activities against Gram-positive strains or they are more potent. The reason may be the difference in cell wall structure, since G+ bacteria have thick peptidoglycan membranes lacking in G- bacteria. All compounds, except for **L1**, are less effective against *C. albicans* than against the bacterial strains. Results show weak activity for **L1**; however, the reduction of the imine bond in **L11** significantly shifts MIC values to lower concentrations. Therefore, we assume the importance of the single C-NH bond in reduced Schiff bases for higher activity. This might be caused by the higher flexibility of the molecular scaffold after reduction of the double azomethine bond, which could facilitate interactions of the molecule with active sites of enzymes. The same trend was observed by [44], when reducing the imine bond in cyclohexane-1,2-diamine derivatives greatly increased antimicrobial activity. Interestingly, a similar Schiff base ligand *trans*-*N*,*N*-bis[(2,4-dichlorophenyl) methylidene] cyclohexane-1,2-diamine showed no antimicrobial activity in a panel of various microorganisms, whereas its corresponding copper, nickel, and palladium complexes exerted moderate activities [45]. This indicates that the presence of the fluorine substituents on the benzene ring clearly facilitates antimicrobial activity.

The highest activity in our tested compounds was observed in the **Zn-L12** complex (MIC 0.0008 µM) with a -CF_3_ group in the para position of the benzene ring. Ligand **L12** has also significantly higher activity compared to **L11.** The activity of the zinc complex with the highest activity against both *S. aureus* and *E. coli* (**Zn-L12**) was significantly higher than the activity of its ligand **L12**; hence, the activity of the free ligand was improved as a result of complexation with the metal. Lipophilicity of the compounds plays an important role in antimicrobial activity as only lipid-soluble substance can pass through the lipid cell wall of bacteria. Coordination of the ligand to a metal ion can also increase the lipophilicity of the compounds via delocalization of the π electrons over the whole chelate ring and via reduction in the polarity of the metal ion due to the overlap of the ligand orbital and partial sharing of the positive charge of the metal ion with donor groups [46].

## 4. Conclusions

We prepared and characterized four reduced Schiff base ligands and their zinc(II) complexes. All compounds were characterized by X-ray crystallography and evaluated against bacterial strains *S. aureus, E. coli* and fungal *C. albicans*. The complex **Zn-L12** showed very good antimicrobial activity against *S. aureus*. The reason for the high activity of this particular derivative (with a trifluoromethyl group in the *para*-position of benzene ring) is unknown and further research to clarify the structure–activity relationships is needed. Additional testing of biological activities is underway and will be published in due course.

Several analogous zinc complexes have been reported in the literature, with the general structure Zn(L)Cl_2_, where L is a reduced Schiff base ligand formed from *trans*-1,2-cyclohexanediamine and variously substituted benzaldehydes or similar aldehydes, as described in the Introduction. Unfortunately, no biological activities have been published so far. We have not been able to prepare zinc(II) complexes with the non-reduced Schiff bases. The low stability of zinc complexes with these ligands might be a possible reason for the lack of reports on such complexes in the scientific literature. Only the preparation of Cu(II), Ni(II), and Pd(II) complexes with *trans*-*N*,*N′*-bis[(2,4-dichlorophenyl) methylidene] cyclohexane-1,2-diamine has been described, although without the X-ray crystal structure analysis of the metal complexes [45].

## Figures and Tables

**Table 1:** Analytical and physical characterisation of prepared ligands **L1**–**L4**, **L11**–**L14**, and complexes **Zn-L11**–**Zn-L14**.

Compound (Chemical Formula)	MW (g/mol)	Color	M.p. °C	Yield (%)	Elemental Analysis Found (Calcd) (%)		
					C	H	N
**L1** (C_20_H_20_F_2_N_2_)	326.38	white	128–129	59.5	72.86 (73.60)	5.77 (6.18)	8.66 (8.58)
**L2** (C_22_H_20_F_6_N_2_)	426.40	white	100–101	86.4	61.05 (61.97)	3.31 (4.73)	6.39 (6.57)
**L3** (C_20_H_18_F_4_N_2_)	362.36	white	103–105	95.0	65.43 (66.29)	4.26 (5.01)	7.57 (7.73)
**L4** (C_24_H_18_F_12_N_2_)	562.39	white	112–113	66.5	53.64 (51.26)	2.72 (3.23)	4.81 (4.98)
**L11** (C_20_H_26_Cl_2_F_2_N_2_)	403.34	white	232–235	56.2	58.99 (59.56)	6.30 (6.50)	7.02 (6.95)
**L12** (C_22_H_26_Cl_2_F_6_N_2_)	503.35	white	240–245	52.6	50.21 (52.50)	4.81 (5.21)	5.91 (5.57)
**L13** (C_20_H_24_Cl_2_F_4_N_2_)	439.32	white	202–207	53.3	52.84 (54.68)	4.77 (5.51)	6.55 (6.38)
**L14** (C_24_H_24_Cl_2_F_12_N_2_)	639.35	white	239–242	44.3	44.81 (45.09)	2.93 (3.78)	4.43 (4.38)
**Zn-L11** (C_20_H_24_Cl_2_ZnF_2_N_2_)	466.70	white	283–284	82.6	51.11 (51.47)	4.70 (5.18)	5.96 (6.00)
**Zn-L12** (C_22_H_24_Cl_2_ZnF_6_N_2_)	566.72	white	292–293	85.7	45.77 (46.63)	3.52 (4.27)	4.88 (4.94)
**Zn-L13** (C_20_H_22_Cl_2_ZnF_4_N_2_)	502.68	white	285–286	78.6	44.05 (47.79)	3.49 (4.41)	5.02 (5.57)
**Zn-L14** (C_24_H_22_Cl_2_ZnF_12_N_2_)	702.71	white	290–291	80.0	43.01 (41.02)	2.50 (3.16)	5.16 (3.99)

**Table 2:** Selected bond lengths (Å) and angles (°) for the complexes.

Bond Lengths in Å		Bond Angles in °	
**Zn-L11**			
Zn1–Cl2	2.2330(4)	Cl1–Zn1–Cl2	120.118(19)
Zn1–Cl1	2.2093(4)	N1–Zn1–Cl2	107.32(4)
Zn1–N1	2.0723(14)	N1–Zn1–Cl1	114.34(4)
Zn1–N2	2.0594(14)	N2–Zn1–Cl2	108.59(4)
		N2–Zn1–Cl1	113.74(4)
		N2–Zn1–N1	88.02(5)
**Zn-L12**			
Zn1–Cl1	2.2165(6)	Cl1–Zn1–Cl2	117.30(2)
Zn1–Cl2	2.2171(6)	N1–Zn1–Cl2	109.66(5)
Zn1–N1	2.0699(18)	N1–Zn1–Cl1	113.28(5)
Zn1–N2	2.0701(18)	N2–Zn1–Cl2	113.70(5)
		N2–Zn1–Cl1	111.80(5)
		N2–Zn1–N1	87.31(7)
**Zn-L13**			
Zn1–Cl1	2.2278(5)	Cl1–Zn1–Cl1^i^	118.83(3)
Zn1–Cl1^i^	2.2278(5)	N1^i^–Zn1–Cl1	108.83(4)
Zn1–N1^i^	2.0752(16)	N1–Zn1–Cl1^i^	108.83(4)
Zn1–N1	2.0752(16)	N1–Zn1–Cl1	114.41(5)
		N1^i^–Zn1–Cl1^i^	114.41(5)
		N1^i^–Zn1–N1	87.35(8)
**Zn-L14**			
Zn1–Cl1	2.2265(6)	Cl1–Zn1–Cl1	109.74(3)
Zn1–Cl1^ii^	2.2265(6)	N1^ii^–Zn1–Cl1	119.55(5)
Zn1–N1^ii^	2.0683(18)	N1–Zn1–Cl1^ii^	119.55(5)
Zn1–N1	2.0683(19)	N1–Zn1–Cl1	110.12(5)
		N1^ii^–Zn1–Cl1^ii^	110.12(5)
		N1^ii^–Zn1–N1	86.66(10)

**Table 3:** Antimicrobial activities MIC (mM) of tested compounds.

Compound	*S. aureus*	*E. coli*	*C. albicans*
**L1**	5.7063	5.7063	2.8531
**L11**	0.7083	0.3541	2.8331
**L12**	0.0028	0.0221	0.3536
**Zn-L11**	0.1076	0.2151	1.7206
**Zn-L12**	0.0008	0.0134	0.8550
**Zn-L13**	0.1054	0.2107	1.6856
**Zn-L14**	0.1063	0.1063	1.7006
**cipro**	0.00068	<0.0003	-

## Data Availability

Not applicable.

## Supplementary Materials

The following supporting information can be downloaded at: https://www.mdpi.com/article/10.3390/life13071516/s1, NMR and IR characterisation of prepared compounds; Figures S1–S11: crystal packing of the prepared compounds; Tables S1–S3: Hydrogen bonds and interactions for the compounds; Tables S4–S6: Crystal data and structure refinement for the compounds.
