# Supplementary material for: Antimicrobially Active Zn(II) Complexes of Reduced Schiff Bases Derived from Cyclohexane-1,2-diamine and Fluorinated Benzaldehydes—Synthesis, Crystal Structure and Bioactivity
Source: Life (Basel). 2023 Jul 6;13(7):1516. doi: 10.3390/life13071516 (PMC10381420; doi:10.3390/life13071516)
Supplement: Supplementary file 1 [file life-13-01516-s001.zip › IR.zip!/Zn-L11.pdf]

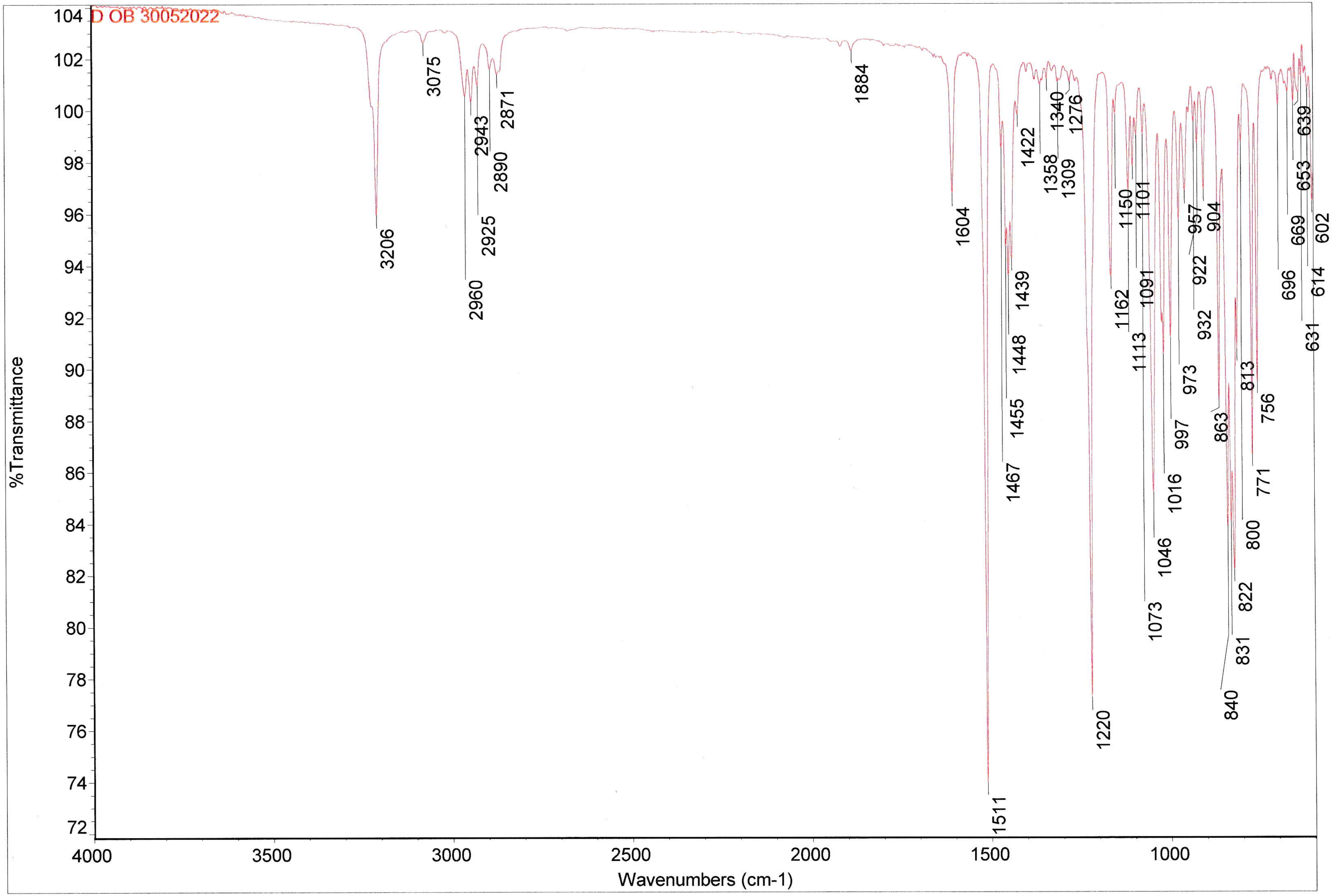

D OB 30052022
%Transmittance
Wavenumbers (cm-1)
3206
3075
2960
2943
2925
2890
2871
1884
1604
1511
1467
1455
1448
1439
1422
1358
1340
1309
1276
1220
1162
1150
1113
1101
1091
1073
1046
1016
997
973
957
932
922
904
863
840
831
822
813
800
771
756
696
669
653
639
631
614
602
