# Supplementary material for: Antimicrobially Active Zn(II) Complexes of Reduced Schiff Bases Derived from Cyclohexane-1,2-diamine and Fluorinated Benzaldehydes—Synthesis, Crystal Structure and Bioactivity
Source: Life (Basel). 2023 Jul 6;13(7):1516. doi: 10.3390/life13071516 (PMC10381420; doi:10.3390/life13071516)
Supplement: Supplementary file 1 [file life-13-01516-s001.zip › IR.zip!/Zn-L12.pdf]

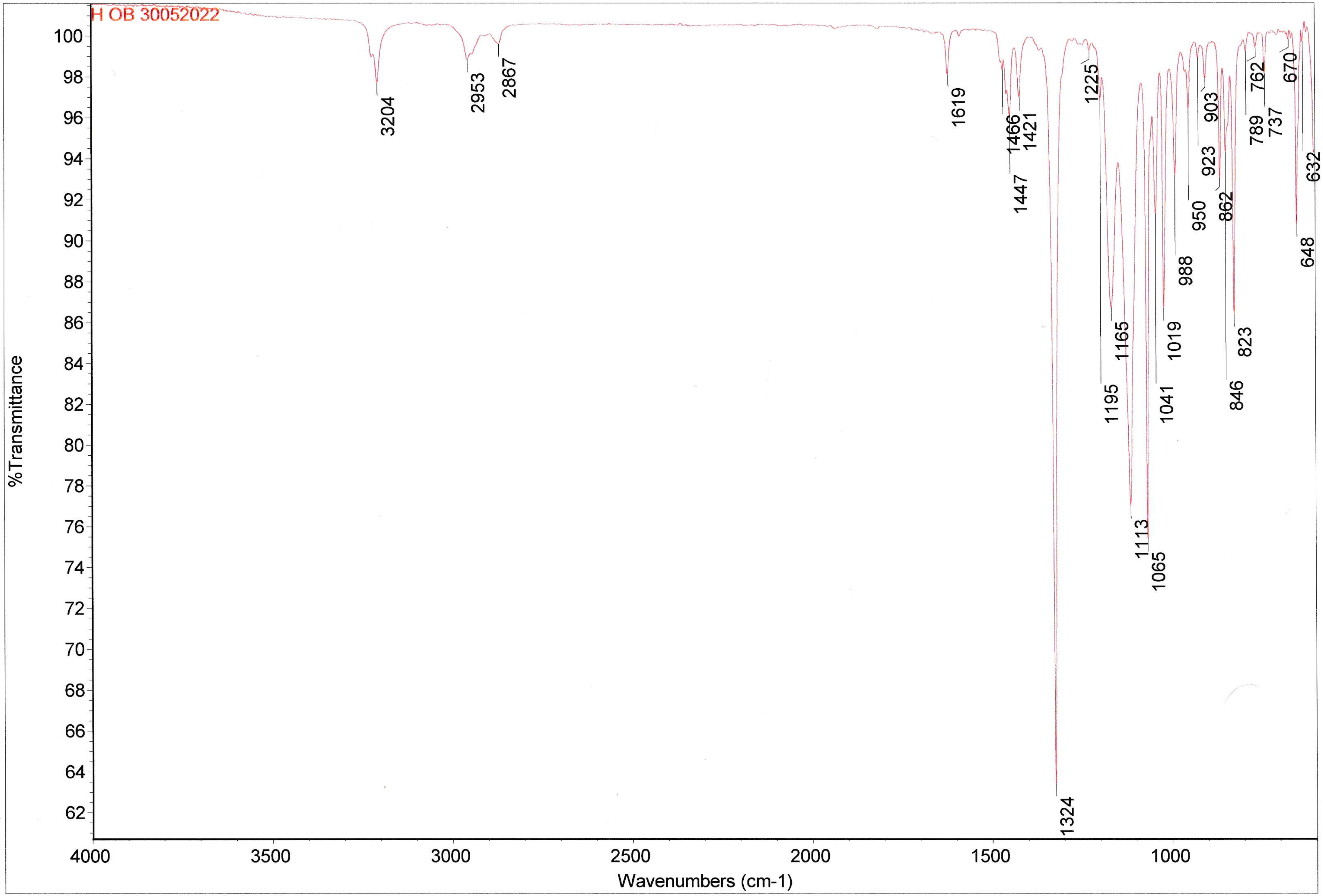
H OB 30052022
%Transmittance
Wavenumbers (cm-1)
100
98
96
94
92
90
88
86
84
82
80
78
76
74
72
70
68
66
64
62
4000
3500
3000
2500
2000
1500
1000
3204
2953
2867
1619
1466
1447
1421
1324
1225
1195
1165
1113
1065
1041
1019
988
950
923
903
862
846
823
789
762
737
670
648
632
