# Supplementary material for: Antimicrobially Active Zn(II) Complexes of Reduced Schiff Bases Derived from Cyclohexane-1,2-diamine and Fluorinated Benzaldehydes—Synthesis, Crystal Structure and Bioactivity
Source: Life (Basel). 2023 Jul 6;13(7):1516. doi: 10.3390/life13071516 (PMC10381420; doi:10.3390/life13071516)
Supplement: Supplementary file 1 [file life-13-01516-s001.zip › IR.zip!/Zn-L13.pdf]

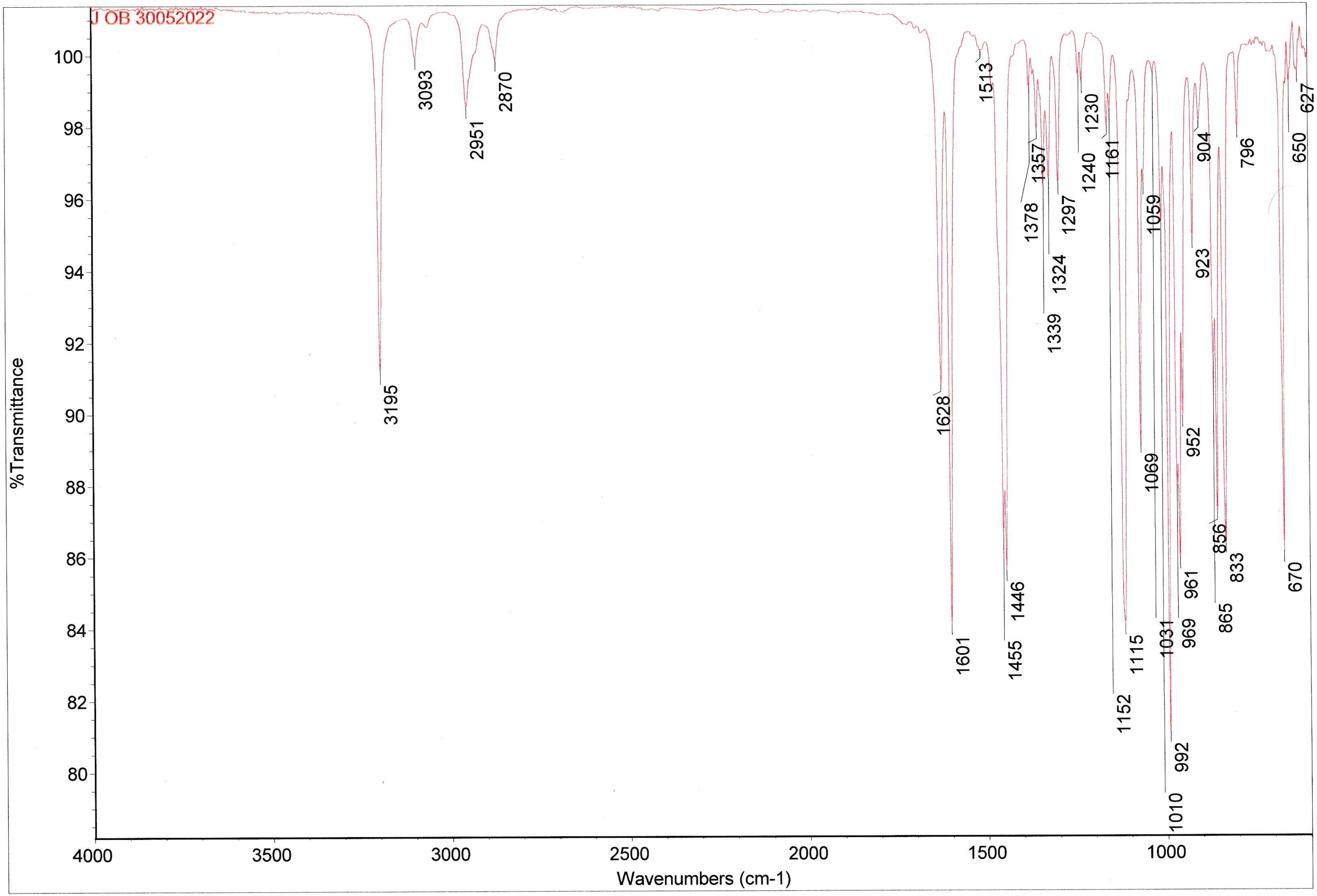
J OB 30052022
%Transmittance
Wavenumbers (cm-1)
100
98
96
94
92
90
88
86
84
82
80
4000
3500
3000
2500
2000
1500
1000
3195
3093
2951
2870
1628
1601
1513
1455
1446
1378
1357
1339
1324
1297
1240
1230
1161
1152
1115
1069
1059
1031
1010
992
969
961
952
923
904
865
856
833
796
670
650
627
