# Supplementary material for: Antimicrobially Active Zn(II) Complexes of Reduced Schiff Bases Derived from Cyclohexane-1,2-diamine and Fluorinated Benzaldehydes—Synthesis, Crystal Structure and Bioactivity
Source: Life (Basel). 2023 Jul 6;13(7):1516. doi: 10.3390/life13071516 (PMC10381420; doi:10.3390/life13071516)
Supplement: Supplementary file 1 [file life-13-01516-s001.zip › IR.zip!/Zn-L14.pdf]

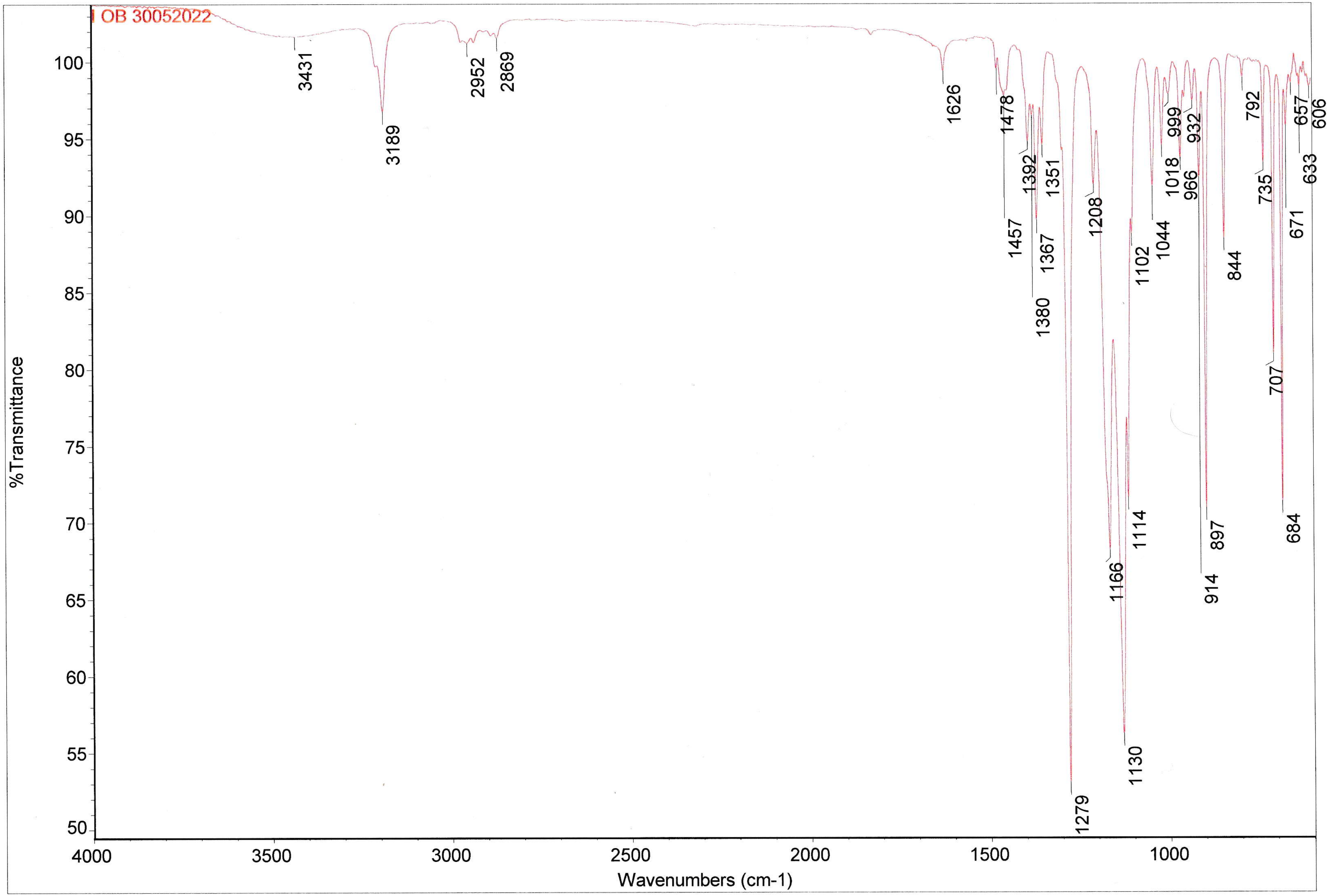
I OB 30052022
%Transmittance
Wavenumbers (cm-1)
100
95
90
85
80
75
70
65
60
55
50
4000
3500
3000
2500
2000
1500
1000
3431
3189
2952
2869
1626
1478
1457
1392
1380
1367
1351
1279
1208
1166
1130
1114
1102
1044
1018
999
966
932
914
897
844
792
735
707
684
671
657
633
606
