# Supplementary material for: Antimicrobially Active Zn(II) Complexes of Reduced Schiff Bases Derived from Cyclohexane-1,2-diamine and Fluorinated Benzaldehydes—Synthesis, Crystal Structure and Bioactivity
Source: Life (Basel). 2023 Jul 6;13(7):1516. doi: 10.3390/life13071516 (PMC10381420; doi:10.3390/life13071516)
Supplement: Supplementary file 1 [file life-13-01516-s001.zip › life-2424373-supplementary-Proof.zip!/life-2424373-supplementary-Proof.pdf]

Supplementary Materials

NMR and IR characterisation of ligands and Zn(II) complexes:

(±)-trans-N,N´-Bis-(4-fluoro-benzylidene)-cyclohexane-1,2-diamine (L1):

IR (neat, cm^{-1}): 2936, 2860 ν(CH_2/CH), 1645 ν(CH=N), 1601, 1506, 1226 ν(C-F), 834. ^{1}H NMR (400 MHz, $CDCl_3$, δ, ppm) 1.45 – 1.51 (m, 2H); 1.74 – 1.87 (m, 6H); 3.33 – 3.40 (m, 2H); 6.96 – 7.01 (m, 4H); 7.54 – 7.59 (m, 4H); 8.13 (s, 2H). ^{13}C-NMR (100 MHz, $CDCl_3$, δ, ppm): 24.44, 32.88 (CH_2); 73.70 (CH); 115.35, 115.55, 129.64, 129.73, 132.55, 132.58 (arom-C); 159.55, 162.77 (CH=N); 165.28 (CF).

(±)-trans-N,N´-Bis-(4-trifluoromethyl-benzylidene)-cyclohexane-1,2-diamine (L2):

IR (neat, cm^{-1}): 2936, 2862 ν(CH_2/CH), 1643 ν(CH=N), 1320 ν(CF), 1170, 1126, 1064, 842. ^{1}H NMR (400 MHz, $CDCl_3$, δ, ppm): 1.48 – 1.53 (m, 2H); 1.78 – 1.90 (m, 6H); 3.44 – 3.61 (m, 2H); 7.56 (d, J = 8.2 Hz, 4H); 7.69 (d, J = 7.0 Hz, 4H); 8.22 (s, 2H). ^{13}C-NMR (100 MHz, $CDCl_3$, δ, ppm): 24.32, 32,74 (CH_2); 73.87 (CH); 122.52 (CF_3); 125.42, 125.22, 125.35, 125.39, 125.42, 125.46, 127.93, 128.04, 131.79, 132,11, 139.33 (arom-C); 159.43 (CH=N).

(±)-trans-N,N´-Bis-(3,5-difluorobenzylidene)-cyclohexane-1,2-diamine (L3):

IR (neat, cm^{-1}): 3093, 3055, 3021, 2938, 2862 ν(CH_2/CH), 1648 ν(CH=N), 1622, 1594, 1318, 1119 ν(CF), 854, 670. ^{1}H NMR (400 MHz, $CDCl_3$, δ, ppm): 1.45 – 1.51 (m, 2H); 1.75 – 1.89 (m, 6H); 3.36 – 3.43 (m, 2H); 6.76 – 6.81 (m, 2H); 7.09 – 7.15 (m, 4H); 8.08 (s, 2H). ^{13}C-NMR (100 MHz, $CDCl_3$, δ, ppm): 24.25, 32.65 (CH_2); 73.54 (CH); 105.33, 105.59, 105.84, 110.34, 110.41, 110.53, 110.60, 139.42, 139.51, 139.60, 158.33, 158.36, 158.39 (arom-C); 161.72, 161.84 (CH=N); 164.20, 164.32 (CF).

(±)-trans-N,N´-Bis-(3,5-bis-trifluoromethylbenzylidene)-cyclohexane-1,2-diamine (L4):

IR (neat, cm^{-1}): 2942, 2872 ν(CH_2/CH), 1652 ν(CH=N), 1277 ν(CF), 1131, 894, 681. ^{1}H NMR (400 MHz, $CDCl_3$, δ, ppm): 1.49 – 1.62 (m, 2H), 1.80 – 1.92 (m, 6H); 3.46 – 3.53 (m, 2H); 7.83 (s, 2H); 8.04 (s, 4H); 8.27 (s, 2H). ^{13}C-NMR (100 MHz, $CDCl_3$, δ, ppm): 24.20, 32.60 (CH_2); 73.76 (CH); 118.96, 121.67, 123.69, 123.73, 123.77 (CF_3); 124.39, 127.10, 127.64, 127.67, 131.50, 131.83, 132.16, 132.50, 138.01 (arom-C); 157.70 (CH=N).

(±)-trans-N,N´-Bis-(4-fluorobenzyl)-cyclohexane-1,2-diaminium dichloride (L11):

IR (neat, cm^{-1}): 2937 ν(CH_2/CH), 2702, 2630 ν (NH_2^+), 1603, 1509, 1221 ν(CF), 833. ^{1}H NMR (400 MHz, CD_3OD, δ, ppm): 1.42 – 1.47 (m, 2H); 1.67 (d, J = 11.0 Hz, 2H); 1.89 – 1.92 (m, 2H); 2.47 (d, J = 13.1 Hz, 2H); 3.55 (s, 2H); 4.29 (d, J = 13.0 Hz, 2H); 4.40 (d, J = 13.0 Hz, 2H); 7.16 – 7.22 (m, 4H); 7.68 – 7.71 (m, 4H). ^{13}C-NMR (100 MHz, CD_3OD, δ, ppm): 22.23, 26.37 (CH_2); 57.01 (CH); 115.50, 115.72, 132.53, 132.61 (arom-C); 162.26, 164.73 (CF).

(±)-trans-N,N´-Bis-(4-trifluoromethylbenzyl)-cyclohexane-1,2-diaminium dichloride (L12):

IR (neat, cm^{-1}): 2934, 2873 ν(CH_2/CH), 2707, 2630 ν(NH_2^+), 1621, 1323 ν (CF), 1162, 1127, 1067, 828. ^{1}H NMR (400 MHz, CD_3OD, δ, ppm): 1.43 – 1.48 (m, 2H); 1.69 – 1.72 (m, 2H); 1.92 – 1.94 (m, 2H); 2.51 (d, J = 15.3 Hz, 2H); 3.62 (s, 2H); 4.39 (d, J = 13.1 Hz, 2H); 4.53 (d, J = 13.1 Hz, 2H); 7.76 (d, J = 8.2 Hz, 4H); 7.88 (d, J = 8.0 Hz, 4H). ^{13}C-NMR (100 MHz, CD_3OD, δ, ppm): 22.38, 26.53 (CH_2); 57.62 (CH); 122.56 (CF_3); 125.26, 125.49, 125.53, 125.60, 126.64, 130.76, 130.86, 131.07, 131.40, 135.06 (arom-C).

(±)-trans-N,N´-Bis-(3,5-difluorobenzyl)-cyclohexane-1,2-diamininium dichloride (L13):

IR (neat, cm^{-1}): 3046, 2950, 2874 ν(CH_2/CH), 2629, 2593 ν(NH_2^+), 1625, 1597, 1459, 1321, 1124 ν(CF), 858, 698. ^{1}H NMR (400 MHz, CD_3OD, δ, ppm): 1.41 – 1.46 (m, 2H); 1.63 – 1.65 (m, 2H); 1.90 – 1.93 (m, 2H); 2.46 (d, J = 13.2 Hz, 2H); 3.51 (s, 2H); 4.30 (d, J = 13.1 Hz, 2H); 4.44 (d, J = 13.2 Hz, 2H); 7.04 – 7.09 (m, 2H); 7.34 (d, J = 5.8 Hz, 4H). ^{13}C-NMR (100 MHz, CD_3OD, δ, ppm): 22.42, 26.63 (CH_2); 57.66 (CH); 104.51, 112.96, 113.20 (arom-C); 161.87, 162.00, 164.35, 164.48 (CF).

(±)-trans-N,N´-Bis-(3,5-bis-trifluoromethylbenzyl)-cyclohexane-1,2-diaminium dichloride (L14):

IR (neat, cm^{-1}): 2955 ν(CH_2/CH), 2725, 2563, 2408 ν(NH_2^+), 1597, 1278 ν(CF), 1139, 907, 683. ^{1}H NMR (400 MHz, CD_3OD, δ, ppm): 1.45 – 1.50 (m, 2H); 1.69 (s, 2H); 1.94 – 1.97 (m, 2H); 2.52 (d, J = 13.2 Hz, 2H); 3.59 (s, 2H); 4.46 (d, J = 13.1 Hz, 2H); 4.64 (d, J = 13.1 Hz, 2H); 8.06 (s, 2H); 8.33 (s, 4H). ^{13}C-NMR (100 MHz, CD_3OD, δ, ppm): 23.71, 28.42 (CH_2); 59.46 (CH); 119.15, 121.85 (CF_3); 124.56, 127.26, 129.55, 131.27, 131.60, 131.93, 132.26 (C-arom.).

Dichloro[(±)-trans-N,N´-bis-(4-fluorobenzyl)-cyclohexane-1,2-diamine]zinc(II) (Zn-L11):

IR (neat, cm^{-1}): 3206 ν(NH), 2960 – 2871 ν(CH_2/CH), 1604, 1511, 1220 ν(CF), 840. ^{1}H NMR (400 MHz, DMSO-d_6, δ, ppm): 1.13 – 1.35 (m, 4H), 1.71 – 1.73 (m, 4H), 2.30 – 2.43 (m, 2H), 3.71 (t, J = 11.0 Hz, 2H), 3.94 (d, *J* = 12.2 Hz, 2H), 7.13 – 7.17 (m, 4H), 7.53 (s, 4H).). ^{13}C-NMR (100 MHz, DMSO-d_6, δ, ppm): 24.16, 28.38, 48,67 (CH_2); 60.88 (CH); 114.63, 114.84, 131.46, 132.62 (C-arom.); 160.28, 162.70 (CF).

Dichlorido[(±)-trans-N,N´-bis-(4-trifluoromethylbenzyl)-cyclohexane-1,2-diamine]zinc(II) (Zn-L12):

IR (neat, cm^{-1}): 3204 ν(NH), 2953, 2867 ν(CH_2/CH), 1324 ν(CF), 1113, 1065, 823. ^{1}H NMR (400 MHz, DMSO-d_6, δ, ppm): 1.15 – 1.36 (m, 4H); 1.73 – 1.74 (m, 4H); 2.33 – 2.46 (m, 2H); 3.81 (t, J = 11.2 Hz, 2H); 4.08 (d, *J* = 12.6 Hz, 2H); 7.69 (s, 8H). ^{13}C-NMR (100 MHz, DMSO-d_6, δ, ppm): 24.68, 28.96, 49.58 (CH_2); 61.54 (CH); 123.37 (C-F); 125.26, 125.30, 125.38, 126.08, 130.27, 125.34, 126.08, 128.24 – 128.78, 130.27, 141.66 (C-arom.).

Dichlorido[(±)-trans-N,N´-bis-(3,5-difluorobenzyl)-cyclohexane-1,2-diamine]zinc(II) (Zn-L13):

IR (neat, cm^{-1}): 3195 ν(NH), 2951, 2870 ν(CH_2/CH), 1628, 1601, 1446, 1115 ν(CF), 833, 670. ^{1}H NMR (400 MHz, DMSO-d_6, δ, ppm): 1.14 – 1.34 (m, 4H); 1.73 – 1.75 (m, 4H); 2.30 – 2.43 (m, 2H); 3.70 (t, J = 10.2 Hz, 2H); 4.04 (d, J = 12.9 Hz, 2H); 7.11 – 7.22 (m, 6H). ^{13}C-NMR (100 MHz, DMSO-d_6, δ, ppm): 24.64, 28.96, 4.31 (CH_2); 61.49 (CH); 103.06, 103.30, 103.55, 112.67, 141.42 (C-arom.); 161.23, 161.36, 163.67, 163.80 (CF).

Dichlorido[(±)-trans-N,N´-bis-(3,5-bis-trifluoromethylbenzyl)-cyclohexane-1,2-diamine]zinc(II) (Zn-L14):

IR (neat, cm^{-1}): 3189 ν(NH), 2952, 2869 ν(CH_2/CH), 1279 ν(CF), 1166, 1130, 897, 684. ^{1}H NMR (400 MHz, DMSO-d_6, δ, ppm): 1.19 – 1.34 (m, 4H); 1.77 (s, 4H); 2.34 – 2.48 (m, 2H); 3.82 (t, J = 11.6 Hz, 2H); 4.24 (s, 2H); 7.99 (s, 2H); 8.16 (s, 4H). ^{13}C-NMR (100 MHz, DMSO-d_6, δ, ppm): 24.67, 29.02, 49.27 (CH_2); 61.93 (CH); 119.75, 121.64 (CF_3); 122.46, 125.17, 127.89, 129.84, 130.17, 130.49, 130.71, 130.82, 140.09 (C-arom.).

Table S1 Hydrogen bonds and interactions for compounds **L1** – **L4**.

Hydrogen Bonds for **L1**						
**D**	**H**	**A**	**d(D-H)/Å**	**d(H-A)/Å**	**d(D-A)/Å**	**D-H-A/°**
C9	H9B	F1$^{(i)}$	0.99	2.60	3.116(3)	112.5

C30	H30B	F4(ii)	0.99	2.62	3.104(3)	110.5
Hydrogen Bonds for **L2**						
**D**	**H**	**A**	**d(D-H)/Å**	**d(H-A)/Å**	**d(D-A)/Å**	**D-H-A/°**
C10	H10A	F6(iii)	0.99	2.68	3.395(4)	129.8
C11	H11A	F5(iv)	0.99	2.62	3.337(5)	129.1
Hydrogen Bonds for **L3**						
	**H**	**A**	**d(D-H)/Å**	**d(H-A)/Å**	**d(D-A)/Å**	**D-H-A/°**
C32	H32	F4	0.95	2.62	3.0856(13)	110.6
C46	H46	N1(v)	0.95	2.56	3.4900(15)	167.3
C13	H13A	F3(vi)	0.95	2.60	3.2856(14)	126.2
Hydrogen Bonds for **L4**						
**D**	**H**	**A**	**d(D-H)/Å**	**d(H-A)/Å**	**d(D-A)/Å**	**D-H-A/°**
C3	H3	F1(vii)	0.95	2.63	3.531(4)	157.9
C7	H7	F12(viii)	0.95	2.66	3.598(4)	171.4
C13	H13B	F8(ix)	0.99	2.63	3.461(4)	141.9
C15	H15A	F11(x)	0.99	2.56	3.487(4)	155.2
C15	H15B	F1(xi)	0.99	2.60	3.436(4)	142.3
C18	H18	F7(xii)	0.95	2.61	3.193(4)	119.7

Symmetry transformations used to generate equivalent atoms:

(i)-1/2+x,1/2-y,+z; (ii)1/2+x,-5/2-y,+z; (iii)1/2+x,1-y,+z; (iv)1/2-x,-1+y,-1/2+z; (v)1-x,1/2+y,3/2-z; (vi)-x,1-y,1-z; (vii)1-x,1-y,-z; (viii)x,-1+y,+z; (ix)2-x,1-y,1-z; (x)1+x,+y,+z; (xi)1-x,1-y,1-z

Table S2. Hydrogen bonds and interactions for compounds **L11 – L13**.

Hydrogen Bonds for **L11**						
**D**	**H**	**A**	**d(D-H)/Å**	**d(H-A)/Å**	**d(D-A)/Å**	**D-H-A/°**

N1	H1A	Cl2	0.91	2.34	3.106(3)	142
N1	H1B	Cl1	0.91	2.15	3.057(2)	176
N2	H2A	Cl2	0.91	2.35	3.107(2)	141
N3	H2B	Cl1	0.91	2.35	3.127(2)	168
Hydrogen Bonds for **L12**						
**D**	**H**	**A**	**d(D-H)/Å**	**d(H-A)/Å**	**d(D-A)/Å**	**D-H-A/°**
N11	H11A	Cl4	0.91	2.18	3.0716(19)	165
N11	H11B	Cl2	0.91	2.26	3.092(2)	151
N23	H23A	Cl3	0.91	2.26	3.1466(19)	166
N23	H23B	Cl2	0.91	2.43	3.215(2)	144
N41	H41A	Cl4	0.91	2.35	3.172(2)	151
N41	H41B	Cl1	0.91	2.34	3.171(2)	151
N53	H53A	Cl1	0.91	2.34	3.120(2)	144
N53	H53B	Cl3	0.91	2.17	3.077(2)	177
Hydrogen Bonds for **L13**						
**D**	**H**	**A**	**d(D-H)/Å**	**d(H-A)/Å**	**d(D-A)/Å**	**D-H-A/°**
N1	H1A	Cl1	0.91	2.49	3.2302(10)	138
N1	H1B	Cl1	0.91	2.16	3.0703(11)	173

Table S3. Hydrogen bonds and interactions for compounds **Zn-L11 – Zn-L14**.

Hydrogen Bonds for **Zn-L11**						
**D**	**H**	**A**	**d(D-H)/Å**	**d(H-A)/Å**	**d(D-A)/Å**	**D-H-A/°**
C10	H10	F1(i)	0.95	2.589(2)	3.193(2)	121.72(5)
C11	H11	F1(i)	0.95	2.540(2)	3.160(2)	123.06(5)

C13	H13	F1(ii)	0.95	2.506(2)	3.279(2)	138.63(5)
Hydrogen Bonds for **Zn-L12**						
**D**	**H**	**A**	**d(D-H)/Å**	**d(H-A)/Å**	**d(D-A)/Å**	**D-H-A/°**
N1	H1	Cl1(iii)	1.00	2.55	3.462(2)	152
N2	H2	Cl2(iv)	1.00	2.47	3.410(2)	157
C1	H1A	F6A(iv)	0.99	2.47	3.426(4)	161
C1	H1A	F6B(iv)	0.99	2.53	3.43(2)	151´ 11´ 323
C6	H6	F3(v)	0.95	2.54	3.483(3)	169
Hydrogen Bonds for **Zn-L13**						
**D**	**H**	**A**	**d(D-H)/Å**	**d(H-A)/Å**	**d(D-A)/Å**	**D-H-A/°**
N1	H1	Cl1(vi)	1.00	2.41	3.4001(18)	168
C8	H8	F1(vii)	1.00	2.47	3.225(2)	131
Hydrogen Bonds for **Zn-L14**						
**D**	**H**	**A**	**d(D-H)/Å**	**d(H-A)/Å**	**d(D-A)/Å**	**D-H-A/°**
N1	H1	Cl1	1.00	2.31	3.259(2)	158

Symmetry transformations used to generate equivalent atoms:
(i)-x,1-y,1-z; (ii)+x,3/2-y, -1/2+z; (iii)1-x,1-y,,1-z; (iv)1-x,2-y,1-z; (v)-x,-y,-z; (vi)-x,1-y,1-z; (vii)1/2+x,1-y,z

Table S4. Crystal data and structure refinement for compounds **L1**, **L2**, **L3** and **L4**.

Compound	L1	L2	L3	L4
Empirical formula	$C_{20}H_{20}F_2N_2$	$C_{22}H_{20}F_6N_2$	$C_{20}H_{18}F_4N_2$	$C_{24}H_{18}F_{12}N_2$
Temperature [K]	100(1)	100(1)	100(1)	100(1)
Wavelength [Å]	1.54186	1.54186	1.54184	1.54186
Crystal system	Orthorhombic	Orthorhombic	Monoclinic	Triclinic
Space group	*Pna2₁*	*Pca2₁*	$P2_1/c$	*P-1*
Unit cell dimensions [Å], [°]	a = 12.2541(4)<br>b = 7.8234(2)<br>c = 14.2698(4)	a = 21.2205(2)<br>b = 11.0966(3)<br>c = 8.4451(4)	a = 16.01994(8)<br>b = 16.33377(8)<br>c = 13.72619(7)<br>α = 90	a = 9.2376(2)<br>b = 9.7046(2)<br>c = 15.6329(3)<br>α =105.839(2)

	α, β, γ = 90.0	α, β, γ = 90.0	β = 90.5538(4)<br>γ = 90	β = 92.671(2)<br>γ = 117.377(2)
Formula weight	326.38	426.40	362.36	562.40
Volume [Å^3]	3323.94(11)	1988.61(11)	3591.51(3)	1172.68(5)
Z / Calculated density [Mg/m^3]	8 / 1.304	4 / 1.424	8 / 1.340	2 / 1.593
Absorption coeff. [mm^{-1}]	0.759	1.071	0.924	1.488
F(000)	1376	880	1504.0	568
Crystal size [mm]	0.3 x 0.29 x 0.08	0.24 x 0.13 x 0.08	0.32 x 0.23 x 0.18	0.30 x 0.26 x 0.18
Theta range for data collection	10.316 to 144.256°	7.968 to 144.238°	5.516 to 133.202°	10.542 to 130.148°
Index ranges	-10<=h<=20<br>-7<=k<=6<br>-40<=l<=41	-25<=h<=25<br>-13<=k<=13<br>-10<=l<=5	-19<=h<=19<br>-19<=k<=19<br>-16<=l<=15	-10<=h<=10<br>-11<=k<=5<br>-16<=l<=18
Reflections collected / Independent reflections	33784 / 6372<br>[R(int) = 0.0234]	43994 / 2847 [R(int) = 0.0563]	64208 / 6349<br>[R(int) = 0.0442]	38679 / 3962 [R(int) = 0.0235]
Refinement method	Full-matrix least-squares on F^2			
Data / restraints / parameters	6372 / 1 / 433	2847 / 1 / 272	6349 / 0 / 469	3962 / 0 / 343
Goodness-of-fit on F^2	1.046	1.063	1.029	1.089
Final R indices [I>2σ(I)]	R1 = 0.0415<br>wR2 = 0.1098	R1 = 0.0461<br>wR2 = 0.1238	R1 = 0.0316<br>wR2 = 0.0800	R1 = 0.0585<br>wR2 = 0.1806
R indices (all data)	R1 = 0.0422<br>wR2 = 0.1103	R1 = 0.0498<br>wR2 = 0.1288	R1 = 0.0338<br>wR2 = 0.0813	R1 = 0.0594<br>wR2 = 0.1814
Flack parameter	0.10(12)	0.0(3)	n/a	n/a
Largest diff. peak and hole [e.Å^{-3}]	0.30 and -0.21	0.47 and -0.40	0.193 and -0.206	0.48 and -0.32

Table S5. Crystal data and structure refinement for compounds **L11** – **L13**.

Compound	**L11**	**L12**	**L13**
Empirical formula	$C_{20}H_{26}Cl_2F_2N_2$	$C_{22}H_{26}Cl_2F_6N_2$	$C_{20}H_{24}Cl_2F_4N_2$
Temperature [K]	100(1)	100(1)	100(1)
Wavelength [Å]	1.54184	1.54184	1.54184
Crystal system	Tetragonal	Orthorhombic	Orthorhombic

Space group	$I4_1/a$	$P2_12_12_1$	P*bcn*
Unit cell dimensions [Å], [°]	a = 24.7671(4)<br>b = 24.7671(4)<br>c = 13.7413(3)<br><br>$\alpha = \beta = \gamma = 90$	a = 13.70588(12)<br>b = 17.22503(16)<br>c = 19.47689(18)<br><br>$\alpha = \beta = \gamma = 90$	a = 13.70588(12)<br>b = 17.22503(16)<br>c = 19.47689(18)<br><br>$\alpha = \beta = \gamma = 90$
Formula weight	403.33	503.35	439.31
Volume [Å^3]	8429.1(4)	4598.19(7)	2084.32(5)
Z / Calculated density [Mg/m^3]	16 / 1.271	8 / 1.454	4 / 1.400
Absorption coeff. [mm^{-1}]	2.968	3.098	3.192
F(000)	3392.0	2080.0	912.0
Crystal size [mm]	0.315 x 0.126 x 0.071	0.48 x 0.05 x 0.04	0.16 x 0.11 x 0.08
Theta range for data collection	7.138 to 133.128°	3.224 to 78.898°	3.046 to 78.729°
Index ranges	-24<=h<=18<br>-26<=k<=29<br>-12<=l<=16	-17<=h<=13<br>-21<=k<=21<br>-24<=l<=24	-17<=h<=17<br>-17<=k<=18<br>-13<=l<=11
Reflections collected / Independent reflections	11957 / 3676<br>[R(int) = 0.0511]	49049 / 8119<br>[R(int) = 0.0479]	17820 / 1844<br>[R(int) = 0.0385]
Refinement method	Full-matrix least-squares on F^2		
Data / restraints / parameters	3676 / 0 / 235	8119 / 0 / 577	1844 / 0 / 127
Goodness-of-fit on F^2	1.090	1.036	1.067
Final R indices [$I>2\sigma(I)$]	R1 = 0.0618<br>wR2 = 0.1748	R1 = 0.0240<br>wR2 = 0.0610	R1 = 0.0256<br>wR2 = 0.0683
R indices (all data)	R1 = 0.0715<br>wR2 = 0.1823	R1 = 0.0251<br>wR2 = 0.0615	R1 = 0.0265<br>wR2 = 0.0693
Flack parameter	n/a	-0.002(4)	n/a
Largest diff. peak and hole [e.Å^{-3}]	0.39 and -0.61	0.257 and -0.171	0.385 and -0.174

Table S6. Crystal data and structure refinement for compounds **Zn-L11** – **Zn-L14**.

Compound	Zn-L11	Zn-L12	Zn-L13	Zn-L14
Empirical formula	$C_{20}H_{24}Cl_2F_2N_2Zn$	$C_{22}H_{24}Cl_2F_6N_2Zn$	$C_{20}H_{22}Cl_2F_4N_2Zn$	$C_{24}H_{22}Cl_2F_{12}N_2Zn$
Temperature [K]	100.02(13)	100(1)	99.99(11)	100(1)
Wavelength [Å]	1.54184	1.54184	1.54184	1.54184
Crystal system	Orthorhombic	Triclinic	Monoclinic	Monoclinic
Space group	P*bca*	*P*-1	C2/c	C2/c
Unit cell dimensions [Å], [°]	a = 13.32270(10)<br>b = 11.12120(10)<br>c = 27.4597(2)<br>α = β = γ = 90	a = 9.7407(3)<br>b = 10.7838(3)<br>c = 12.9831(5)<br>α = 109.817(3)<br>β = 95.541(3)<br>γ = 103.759(3)	a = 11.1380(3)<br>b = 14.1925(3)<br>c = 13.9702(3)<br>α = 90<br>β = 111.490(3)<br>γ = 90	a = 27.2981(8)<br>b = 9.8751(2)<br>c = 10.4760(3)<br>α = 90<br>β = 96.848(2)<br>γ = 90
Formula weight	466.68	566.70	502.66	702.70
Volume [$Å^3$]	4068.55(6)	1222.54(7)	2054.83(9)	2803.88(13)
Z / Calculated density [Mg/m^3]	8 / 1.524	2 / 1.539	4 / 1.625	4 / 1.665
Absorption coeff. [mm^{-1}]	4.311	3.942	4.462	3.889
F(000)	1920.0	576.0	1024.0	1408.0
Crystal size [mm]	0.2 x 0.1 x 0.09	0.15 x 0.10 x 0.07	0.14 x 0.11 x 0.07	0.11 x 0.04 x 0.03
Theta range for data collection	6.438 to 157.304°	3.688 to 77.54°	9.226 to 133.19°	6.522 to 157.064°
Index ranges	-15<=h<=16<br>-14<=k<=8<br>-34<=l<=34	-11<=h<=12<br>-13<=k<=13<br>-16<=l<=16	-10<=h<=13<br>-16<=k<=15<br>-16<=l<=16	-32<=h<=33<br>-12<=k<=12<br>-11<=l<=12
Reflections collected / Independent reflections	20503 / 4143<br>[R(int) = 0.0320]	11784 / 4287<br>[R(int) = 0.0292]	5616 / 1793<br>[R(int) = 0.0425]	20109 / 2874<br>[R(int) = 0.0702]
Refinement method	Full-matrix least-squares on F^2			
Data / restraints / parameters	4143 / 0 / 244	4287 / 0 / 307	1793 / 0 / 132	2874 / 0 / 190
Goodness-of-fit on F^2	1.058	1.021	1.060	1.062
Final R indices [I>2σ(I)]	R_1 = 0.0266<br>wR_2 = 0.0674	R_1 = 0.0324<br>wR_2 = 0.0800	R_1 = 0.0341<br>wR_2 = 0.0948	R_1 = 0.0426<br>wR_2 = 0.1180

R indices (all data)	R_1 = 0.0282<br>wR_2 = 0.0683	R_1 = 0.0344<br>wR_2 = 0.0814	R_1 = 0.0359<br>wR_2 = 0.0960	R_1 = 0.0475<br>wR_2 = 0.1216
Flack parameter	n/a	n/a	n/a	n/a
Largest diff. peak and hole [e.Å^{-3}]	0.30 and -0.42	0.825 and -0.401	0.39 and -0.50	0.82 and -0.40

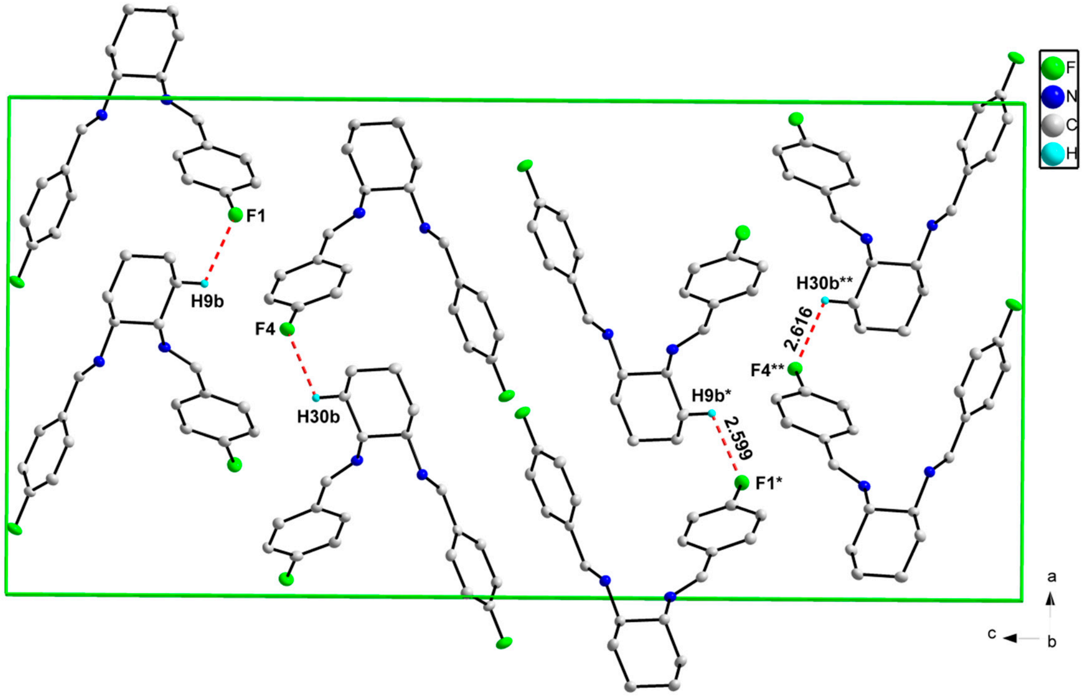

Figure S1. The crystal packing of **L1** viewed along the b axis. Red dashed lines indicate system (intermolecular) hydrogen-bond. Hydrogen atoms are omitted for clarity. Symmetry code: (*) -0.5+x,0.5-y,+z; (**) 0.5+x,-2.5-y,+z.

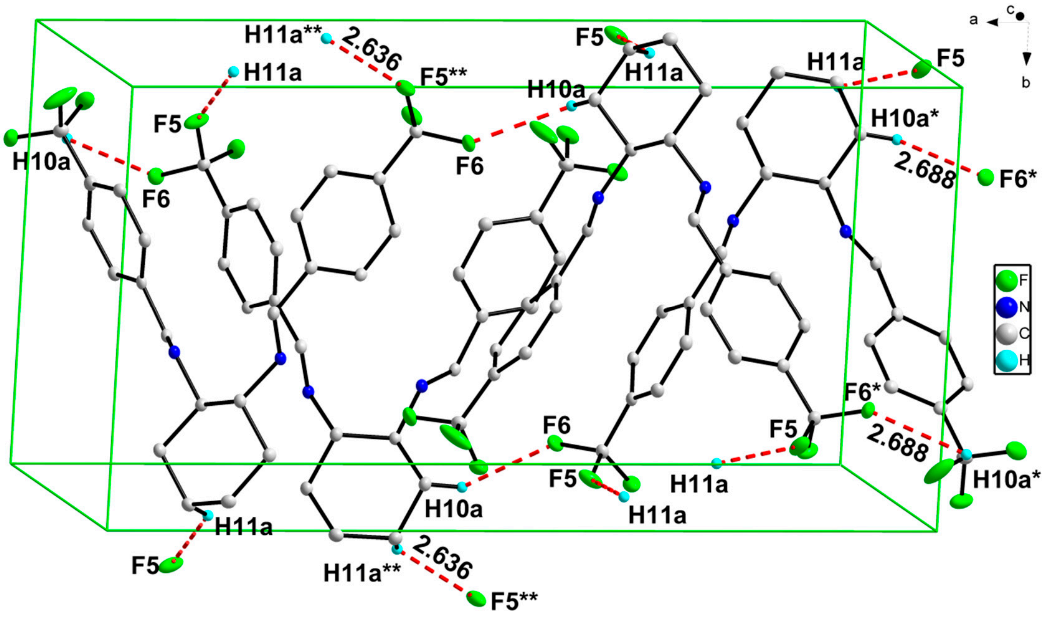

Figure S2. The crystal packing of **L2** viewed between the **a**, **b**, **c** axis. Red dashed lines indicate system (intermolecular) hydrogen-bond. Hydrogen atoms are omitted for clarity. Symmetry code: (*) 0.5+x,1-y,+z; (**) 0.5-x,-1+y,-0.5+z

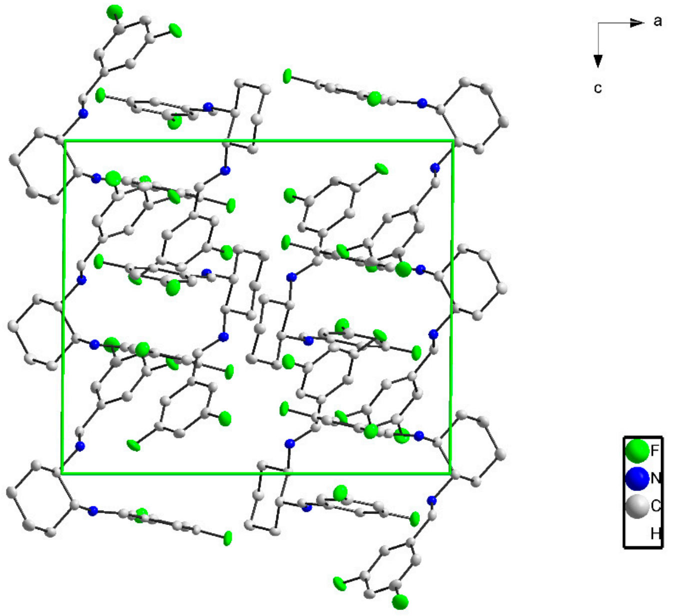

Figure S3. The crystal packing of **L3** viewed between the **a**, **b**, **c** axis. Red dashed lines indicate system (intermolecular) hydrogen-bond. Hydrogen atoms are omitted for clarity.

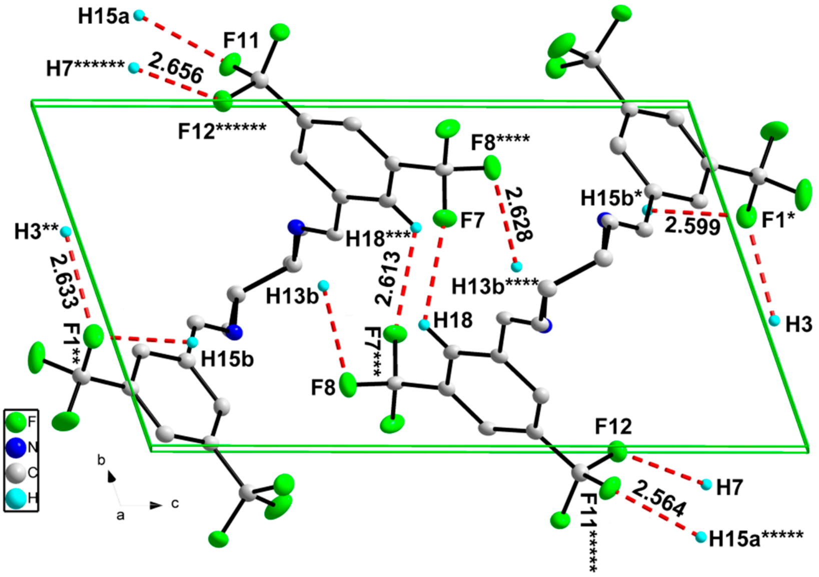

Figure S4. The crystal packing of **L4** viewed along the **a** axis. Red dashed lines indicate system (intermolecular) hydrogen-bond. Hydrogen atoms are omitted for clarity. Symmetry code: (*)1+x,+y,+z; (**)1-x,1-y,-z; (***)1-x,1-y,1-z; (****)2-x,1-y,1-z; (*****)x,-1+y,+z; (******)x,-1+y,+z.

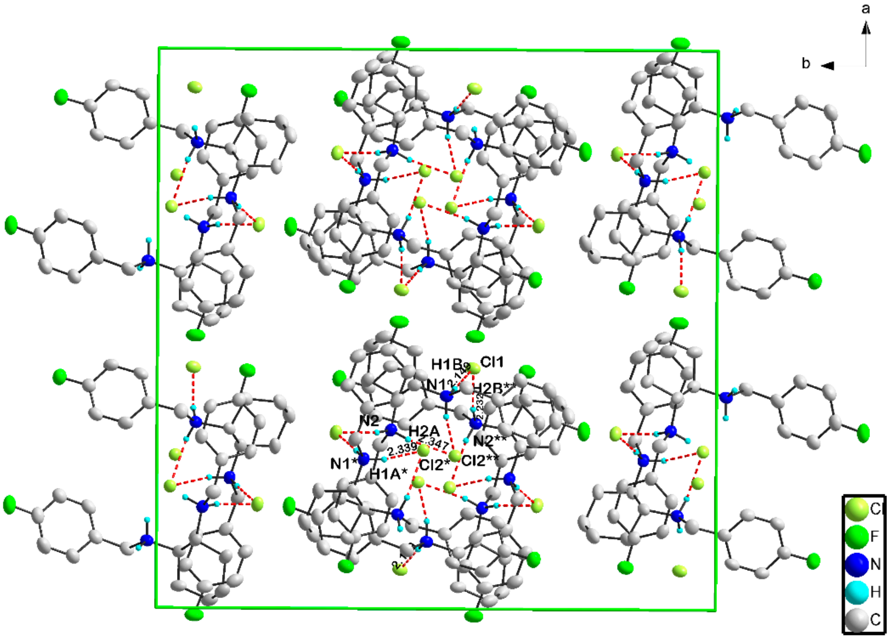

Figure S5. The crystal packing of **L11** viewed between the **a**, **b**, **c** axis. Red dashed lines indicate system (intermolecular) hydrogen-bond. Hydrogen atoms are omitted for clarity. Symmetry code: (*)1+x,+y,+z; (**)1-x,1-y,-z.

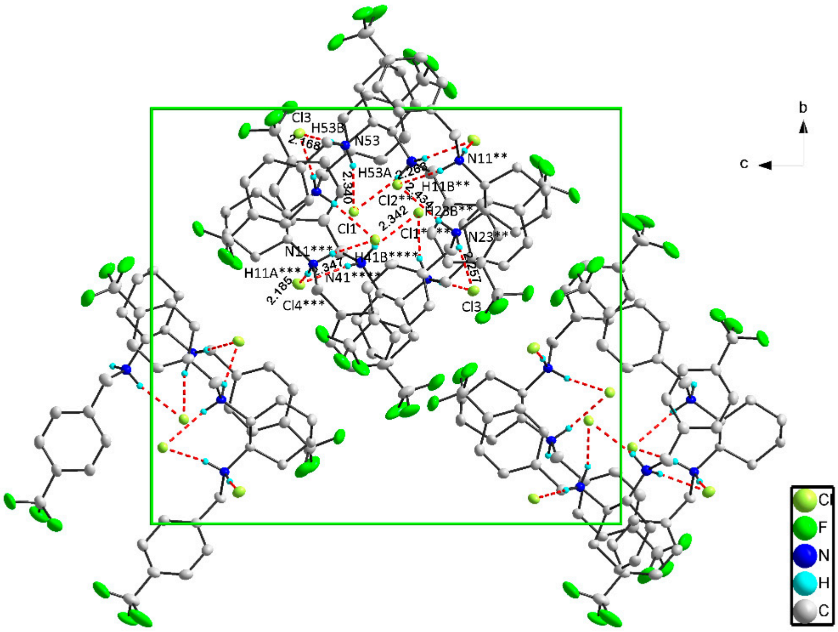

Figure S6. The crystal packing of **L12** viewed between the **a**, **b**, **c** axis. Red dashed lines indicate system (intermolecular) hydrogen-bond. Hydrogen atoms are omitted for clarity. Symmetry code: (*)1+x,+y,+z; (**)1-x,1-y,-z; (***)1-x,1-y,1-z.

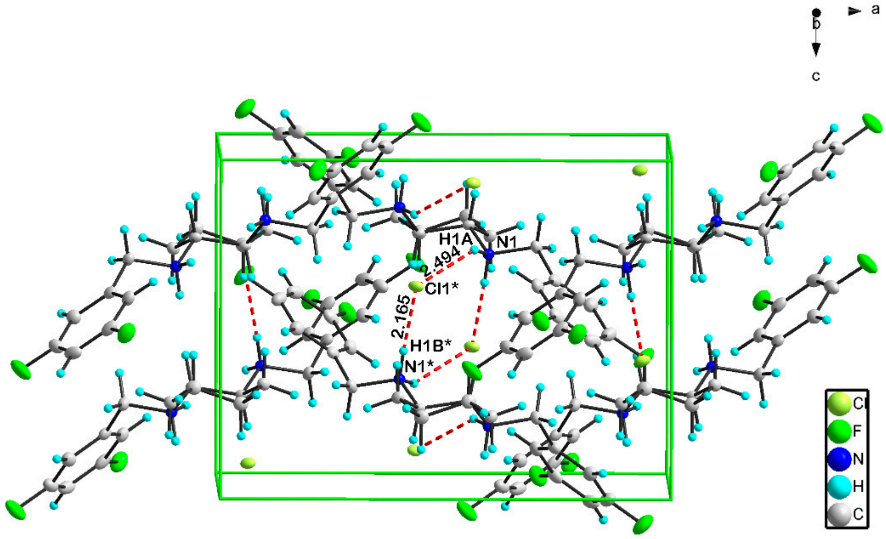

Figure S7. The crystal packing of **L13** viewed between the **a**, **b**, **c** axis. Red dashed lines indicate system (intermolecular) hydrogen-bond. Hydrogen atoms are omitted for clarity. Symmetry code: (*)1+x,+y,+z.

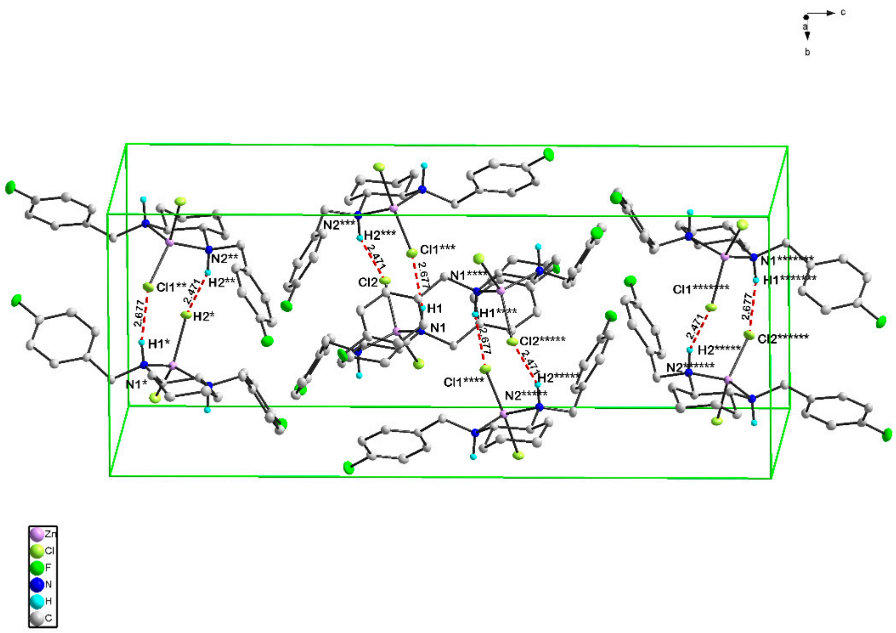

Figure S8. The crystal packing of **Zn-L11** viewed between the **a**, **b**, **c** axis. Red dashed lines indicate system (intermolecular) hydrogen-bond. Hydrogen atoms are omitted for clarity. Symmetry code: (*)1+x,+y,+z; (**)1-x,1-y,-z; (***)1-x,1-y,1-z; (****)2-x,1-y,1-z; (*****)x,-1+y,+z; (******)x,-1+y,+z; (*******)1/2+x,1-y,+z.

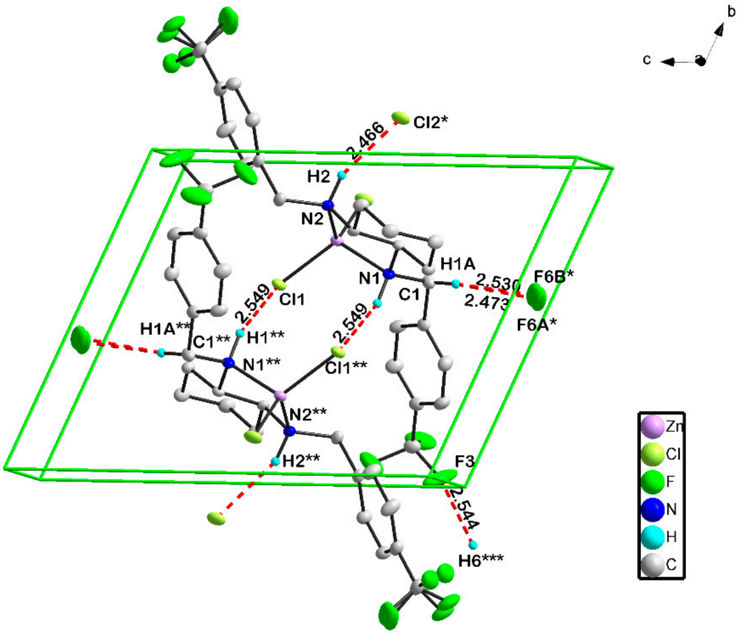

Figure S9. The crystal packing of **Zn-L12** viewed between the **a**, **b**, **c** axis. Red dashed lines indicate system (intermolecular) hydrogen-bond. Hydrogen atoms are omitted for clarity. Symmetry code: (*)1+x,+y,+z; (**)1-x,1-y,-z; (***)1-x,1-y,1-z.

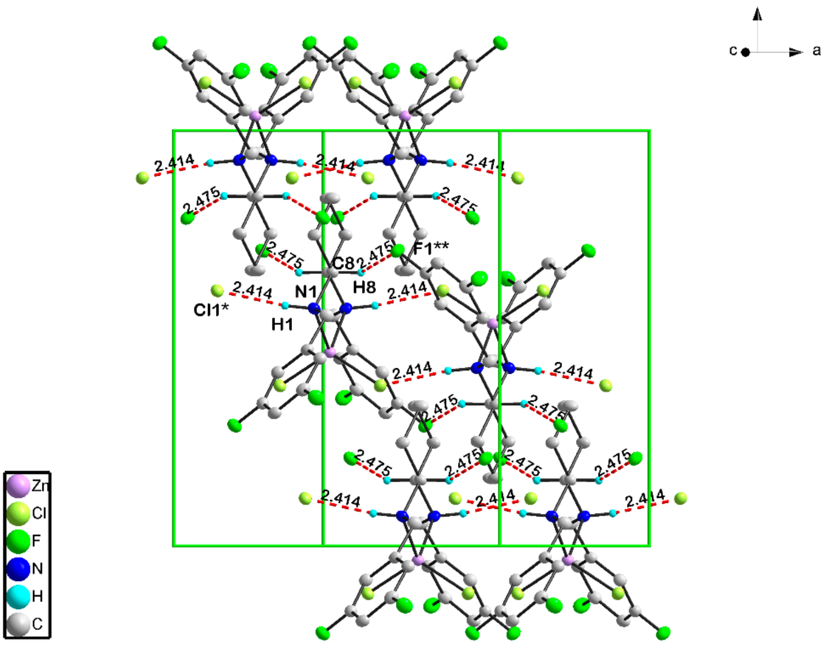

Figure S10. The crystal packing of **Zn-L13** viewed between the **a**, **b**, **c** axis. Red dashed lines indicate system (intermolecular) hydrogen-bond. Hydrogen atoms are omitted for clarity. Symmetry code: (*)1+x,+y,+z; (**)1-x,1-y,-z.

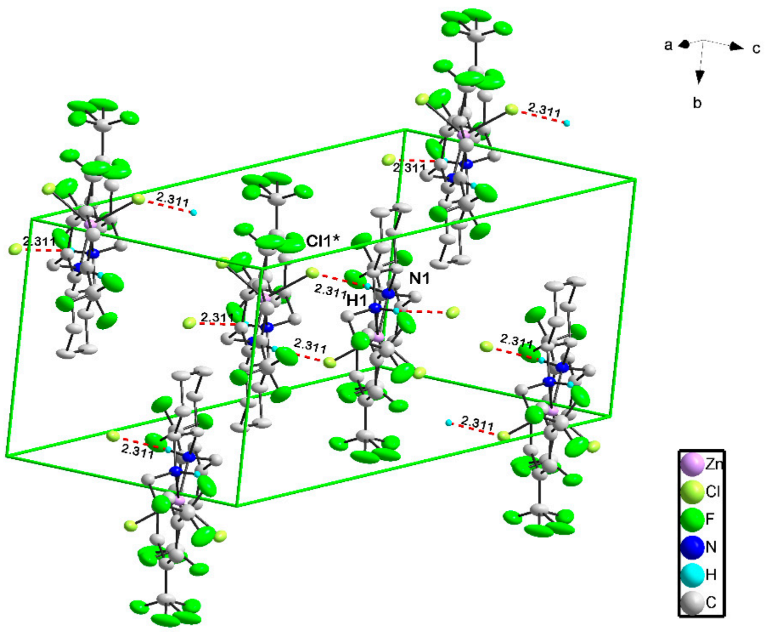

Figure S11. The crystal packing of **Zn-L14** viewed between the **a**, **b**, **c** axis. Red dashed lines indicate system (intermolecular) hydrogen-bond. Hydrogen atoms are omitted for clarity. Symmetry code: (*)1+x,+y,+z.
