# Supplementary material for: Antimicrobially Active Zn(II) Complexes of Reduced Schiff Bases Derived from Cyclohexane-1,2-diamine and Fluorinated Benzaldehydes—Synthesis, Crystal Structure and Bioactivity
Source: Life (Basel). 2023 Jul 6;13(7):1516. doi: 10.3390/life13071516 (PMC10381420; doi:10.3390/life13071516)
Supplement: Supplementary file 1 [file life-13-01516-s001.zip › NMR.zip!/CNMR_Ligand L1.pdf]

13C NMR, 100 MHz, AutoX_DB
BO-2R2
CDCl3
11 Nov 2020

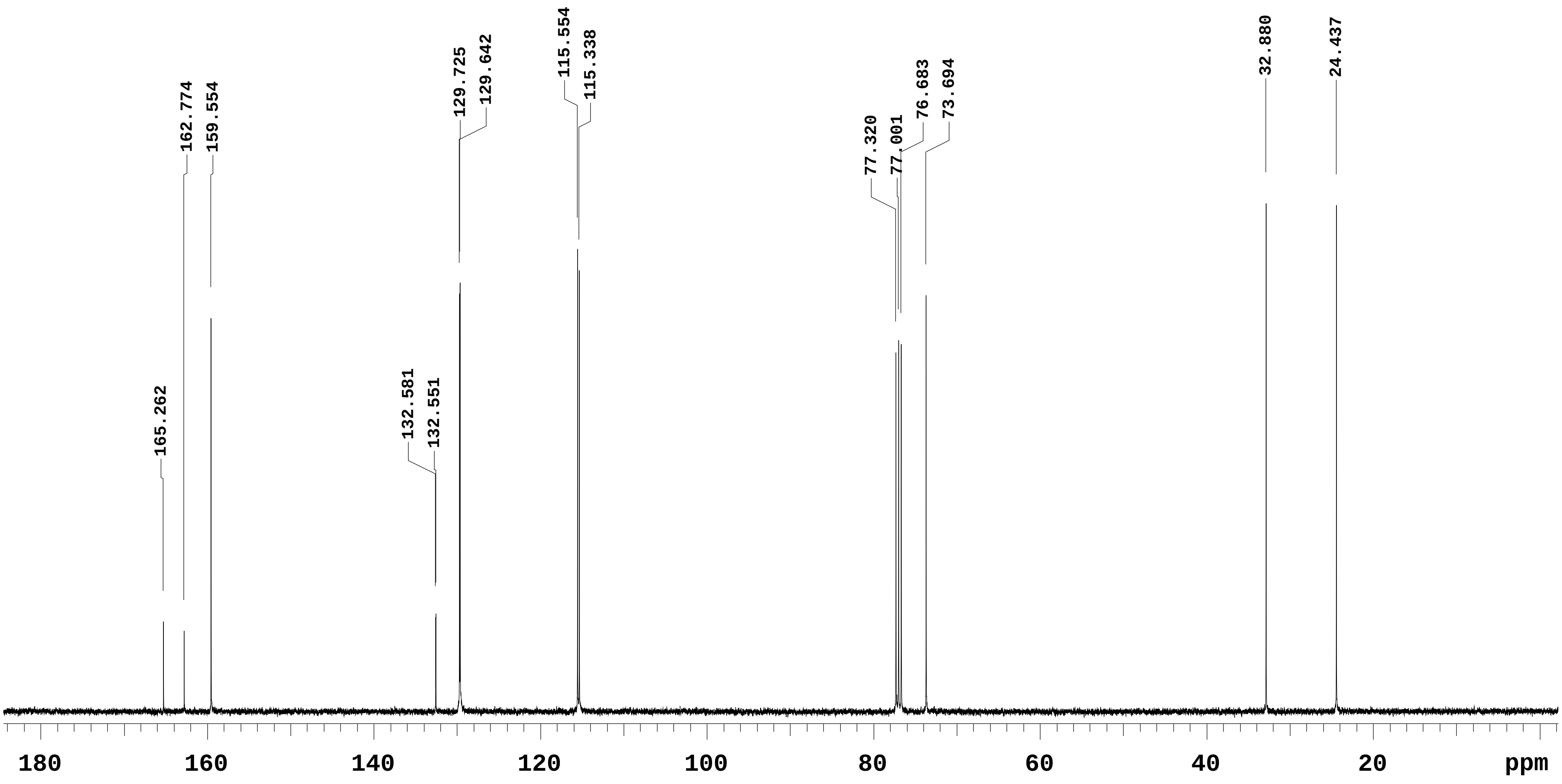


Plotname: CARBON_01_plot01
